# Simulations of Solar and Stellar Dynamos and Their Theoretical Interpretation

**DOI:** 10.1007/s11214-023-01005-6

**Published:** 2023-10-11

**Authors:** Petri J. Käpylä, Matthew K. Browning, Allan Sacha Brun, Gustavo Guerrero, Jörn Warnecke

**Affiliations:** 1 Institute for Astrophysics and Geophysics, University of Göttingen, Friedrich-Hund-Platz 1, Göttingen, 37077 Germany; 2 Leibniz Institute for Solar Physics (KIS), Schöneckstraße 6, Freiburg, 79104 Germany; 3 Department of Physics & Astronomy, University of Exeter, Stocker Road, Exeter, EX4 4QL UK; 4 Département d’Astrophysique/AIM, Univ. Paris-Saclay and Univ. de Paris Cité, CEA, CNRS, Gif-sur-Yvette, 91191 France; 5 Physics Department, Universidade Federal de Minas Gerais, Av. Antonio Carlos 6627, Belo Horizonte, MG 31270-901 Brazil; 6 Physics Department, New Jersey Institute of Technology, 323 Dr Martin Luther King Jr Blvd, Newark, NJ 07103 USA; 7 Max Planck Institute for Solar System Research, Justus-von-Liebig-Weg 3, Göttingen, 37077 Germany

**Keywords:** Dynamo, Magnetohydrodynamics, Simulation, Turbulence

## Abstract

We review the state of the art of three dimensional numerical simulations of solar and stellar dynamos. We summarize fundamental constraints of numerical modelling and the techniques to alleviate these restrictions. Brief summary of the relevant observations that the simulations seek to capture is given. We survey the current progress of simulations of solar convection and the resulting large-scale dynamo. We continue to studies that model the Sun at different ages and to studies of stars of different masses and evolutionary stages. Both simulations and observations indicate that rotation, measured by the Rossby number which is the ratio of rotation period and convective turnover time, is a key ingredient in setting the overall level and characteristics of magnetic activity. Finally, efforts to understand global 3D simulations in terms of mean-field dynamo theory are discussed.

## Introduction

The intriguing coherence of the solar magnetic cycle has fascinated researchers for more than a century starting from Hale’s discovery of magnetic field in sunspots (Hale 1908; Hale et al. 1919), and early attempts to build simple models (Larmor 1919; Cowling 1933). The first successful models of the solar cycle made use of mean-field approximations yielding equations where only the large-scale contributions were explicitly computed, whereas the small scales were characterised by physically plausible parameterizations (Parker 1955; Steenbeck and Krause 1969). Mean-field models opened up new avenues in studying solar and stellar magnetism but their Achilles’ heel is the parameterizations of the small scales which are in general untractable analytically in parameter regimes relevant to stars. This is due to the closure problem of turbulence rendering such models susceptible to fine-tuning. A review of modern mean-field theory is presented elsewhere in this collection (Brandenburg et al. 2023).

Rapidly increasing computing power allowed for the first direct solutions of the equations of (magneto)hydrodynamics in spherical shells in the late 1970s and early 1980s (Gilman 1977; Gilman and Miller 1981; Gilman 1983; Glatzmaier 1985). Prior to the these simulations and the discovery of the internal rotation profile of the Sun, the angular velocity was generally assumed to be constant in cylindrical surfaces and to decrease as a function of radius, in which case the propagation of the dynamo wave from a mean-field $\alpha\Omega$ dynamo is predicted to be equatorward given typical assumptions regarding the influence of the Coriolis force on convective eddies (Parker 1955; Steenbeck and Krause 1969; Yoshimura 1975); see, however, Roberts and Stix (1972). This changed definitively when helioseismology revealed that the angular velocity is actually increasing with radius in the bulk of the solar convection zone (e.g. Duvall et al. 1984; Schou et al. 1998) which lead to the “dynamo dilemma” (Parker 1987). This dilemma was also captured by the early 3D simulations where solar-like differential rotation with fast equator and slow poles was qualitatively reproduced, but where the dynamo waves propagated toward the poles, contrary to the Sun (Gilman 1983; Glatzmaier 1985).

After this, the interest in 3D simulations of solar and stellar dynamos waned and was not rekindled until the early 2000s, starting with the development of the ASH (Anelastic Spherical Harmonic) code (e.g. Miesch et al. 2000; Elliott et al. 2000; Brun and Toomre 2002). While many of the early studies concentrated on the Sun (e.g. Brun et al. 2004; Browning et al. 2006; Miesch et al. 2008), a proliferation of models from various groups using different codes occurred in the 2010s when simulations of more rapidly rotating Suns started to yield cycles and equatorward migration more or less routinely (e.g. Ghizaru et al. 2010; Käpylä et al. 2010; Brown et al. 2011; Käpylä et al. 2012; Nelson et al. 2013; Augustson et al. 2015; Mabuchi et al. 2015; Simitev et al. 2015). Furthermore, simulations of main-sequence stars other than the Sun also started to appear covering the mass range from fully convective M dwarfs (e.g. Dobler et al. 2006; Browning 2008; Yadav et al. 2015; Bice and Toomre 2020; Käpylä 2021) to F stars with thin surface convection zones (Augustson et al. 2013; Breton et al. 2022), as well as core convection, dynamos, and interaction with fossil fields in more massive A, B, and O stars (e.g. Featherstone et al. 2009; Augustson et al. 2016). Models exploring stellar magnetism outside of the main sequence have also started to appear, including pre-main sequence stars (e.g. Emeriau-Viard and Brun 2017), red giants (e.g. Dorch 2004; Brun and Palacios 2009), and newly born neutron stars (e.g. Raynaud et al. 2020; Masada et al. 2022).

Parallel to the developments in simulations, observational data and knowledge regarding stellar magnetism has also experienced explosive growth. We now have dozens of stars with observed cycles from long-term observing campaigns monitoring chromospheric emission (e.g. Baliunas et al. 1995). However, the systematics of these cycles as a function of stellar rotation are still under debate (e.g. Brandenburg et al. 2017; Boro Saikia et al. 2018b; Olspert et al. 2018; Bonanno and Corsaro 2022). Zeeman-Doppler imaging has also revealed polarity reversals (e.g. Kochukhov et al. 2013; Boro Saikia et al. 2018), as well as large-scale non-axisymmetric and dipole-dominated magnetic fields in rapidly rotating late-type stars (e.g. Kochukhov 2021). Finally, magnetic activity saturates when the stellar Rossby number ${\mathrm{Ro}}= P_{\mathrm{rot}}/\tau_{\mathrm{conv}}$, which is the ratio of the rotation period and the convective turnover time, is less than about 0.1, such that for lower ${\mathrm{Ro}}$ the activity and magnetic field strength is roughly constant (e.g. Wright et al. 2018; Reiners et al. 2022). These basic observations are crucial constraints for the numerical simulations. Nevertheless, the Sun still poses the stringest constraints to simulations due its proximity and access to its interior structure through helioseismology. Somewhat surprisingly, the current 3D simulations struggle to reproduce not only the dynamo, but also the convective amplitudes and the differential rotation of the Sun, often yielding anti-solar (slow equator, fast poles) solutions with nominally solar luminosity and rotation rate (e.g. Matt et al. 2011; Käpylä et al. 2014; Gastine et al. 2014; Hotta et al. 2015; Brun et al. 2017). This issue has been dubbed the convective conundrum (O’Mara et al. 2016) and poses arguably the greatest challenge in the field of stellar dynamo simulations today. It has also been suggested that the Sun is close to a transition where its dynamo efficiency diminishes (e.g. van Saders et al. 2016), possibly due to a shift from solar-like to anti-solar differential rotation, making it difficult to capture by simulations (e.g. Käpylä et al. 2014; Brun et al. 2022). Our aim in the following is to review the current successes and shortcomings of current simulations in capturing the relevant observations.

The remainder of the review is organised as follows: the basic equations and physics are discussed in Sect. 2 and the limitations of the numerical approach are reviewed in Sect. 3. The relevant observations and the main results of current 3D simulations of various types of stars are reviewed in Sect. 4 and Sect. 5, respectively. Section 6 gives an overview of the comparisons between mean-field models and global simulations. Finally, we conclude in Sect. 7 with an overview of the state of the field, current challenges, and possible future directions.

## Relevant Physics and Equations

Stellar convection zones are described by the equations of magnetohydrodynamics (MHD), describing the time evolution of the magnetic field and conservation of mass, momentum, and energy:

1 $$\begin{aligned} \frac{\partial \boldsymbol {B}}{\partial t} =& \boldsymbol{\nabla}\times(\boldsymbol {u}\times \boldsymbol {B}- \eta\mu_{0} \boldsymbol {J}), \end{aligned}$$

2 $$\begin{aligned} \frac{\partial\rho}{\partial t} =& - \boldsymbol{\nabla}\boldsymbol{\cdot}(\rho \boldsymbol{u}), \end{aligned}$$

3 $$\begin{aligned} \rho\frac{\partial\boldsymbol{u}}{\partial t} =& - \boldsymbol{\nabla}\boldsymbol{\cdot}(\rho \boldsymbol{u}\boldsymbol{u}) + \rho \boldsymbol {g}- \boldsymbol{\nabla} p - 2 \rho \boldsymbol{\Omega}_{0} \times \boldsymbol {U}+ \boldsymbol {J}\times \boldsymbol {B}+ \boldsymbol{\nabla}\boldsymbol{\cdot} \boldsymbol {F}^{\mathrm{visc}}, \end{aligned}$$

4 $$\begin{aligned} \rho T \frac{\partial s}{\partial t} =& - \boldsymbol{\nabla}\boldsymbol{\cdot}(\rho s \boldsymbol{u}) + \boldsymbol{\nabla}\boldsymbol{\cdot}\boldsymbol{\mathcal{F}} + {\mathcal {H}} + 2\nu\rho\boldsymbol{\mathsf{S}}^{2} + \eta\mu_{0} \boldsymbol {J}^{2}, \end{aligned}$$

where $\boldsymbol {B}$ is the magnetic field, $\boldsymbol{u}$ is the velocity, $\eta$ is the magnetic diffusivity, $\mu_{0}$ is the permeability of vacuum, $\boldsymbol {J}= \mu_{0}^{-1} \boldsymbol{\nabla}\times \boldsymbol {B}$ is the current density, $\rho$ is the fluid density, $\boldsymbol {g}=-\boldsymbol{\nabla}\phi$ is the acceleration due to gravity, where $\phi$ is the gravitational potential, $p$ is the gas pressure, $\boldsymbol{\Omega}_{0}$ is the rotation rate of the star, $\boldsymbol {F}^{\mathrm{visc}}$ is the viscous force, $s$ is the specific entropy, and $\boldsymbol{\mathcal{F}} = \boldsymbol{\mathcal{F}}^{\mathrm{rad}} + \boldsymbol {\mathcal{F}}^{ \mathrm{SGS}}$ describes radiative and any subgrid-scale (SGS) fluxes that are present. ℋ describes additional cooling and heating that is sometimes used instead of, or in addition to, the radiative flux (e.g. Ghizaru et al. 2010; Guerrero et al. 2019; Matilsky et al. 2019), or to take into account heating due to nuclear reactions in the core of the star (e.g. Dobler et al. 2006; Käpylä 2021; Brun et al. 2022). Most often the gas is assumed to be fully ionised and to obey the ideal gas equation $p = {\mathcal{R}}\rho T$, where ${\mathcal{R}}= c_{\mathrm{P}}- c_{\mathrm{V}}$ is the gas constant and $c_{\mathrm{P}}$ and $c_{\mathrm{V}}$ are the specific heat capacities in constant pressure and volume, respectively (see, however, e.g. Hotta et al. 2015; Strugarek et al. 2016, for other approaches).

The viscous force is given by

5 $$\begin{aligned} \boldsymbol{\nabla}\boldsymbol{\cdot} \boldsymbol{F}^{\mathrm{visc}} = \boldsymbol{\nabla}\boldsymbol{\cdot} (2\nu\rho \boldsymbol{\mathsf{S}}) \end{aligned}$$

where $\nu$ is the kinematic viscosity and

6 $$\begin{aligned} \mathsf{S}_{ij}\, = \,{\textstyle{1\over 2}}(U_{i;j} + U_{j;i}) - {\textstyle{1\over 3}}\delta_{ij} \boldsymbol{\nabla}\boldsymbol{\cdot}\boldsymbol{U}, \end{aligned}$$

is the traceless rate of strain tensor where the semicolons denote covariant derivatives (cf. Mitra et al. 2009). In principle $\nu$ is a function of density and temperature according to Spitzer (1962), but in practice the current simulations adapt various physically or numerically motivated formulations that are geared toward minimizing diffusion and maximising numerical stability on a given grid resolution (for a review, see e.g. Miesch et al. 2015).

Due to the short mean-free path of photons in stellar interiors, radiation is typically modeled via the diffusion approximation with

7 $$\begin{aligned} \boldsymbol{\mathcal{F}}^{\mathrm{rad}} = -K \boldsymbol{\nabla} T, \end{aligned}$$

where $K$ is the radiative conductivity which is related to the opacity $\kappa$ of the matter via

8 $$\begin{aligned} K = \frac{16 \sigma_{\mathrm{SB}}T^{3}}{3 \kappa\rho}, \end{aligned}$$

where $\sigma_{\mathrm{SB}}$ is the Stefan–Boltzmann constant. The radiative conductivity is often taken to be a fixed function of radius resulting either from a stellar evolution model (e.g. Brun et al. 2011; Hotta et al. 2022), or a simpler fixed analytic prescription producing a qualitatively similar behavior (e.g. Käpylä et al. 2013; Warnecke 2018). Alternatively, $K$ can also be taken to be dependent on the ambient thermodynamic state in solar-like stars via the Kramers opacity law (e.g. Käpylä et al. 2020; Viviani and Käpylä 2021) with

9 $$\begin{aligned} \kappa\propto\rho T^{-7/2}, \end{aligned}$$

which allows a non-linear back-reaction of, for example, rotation and magnetic fields (e.g. Käpylä et al. 2019). Radiative cooling and heating can also be included via the heating/cooling term,

10 $$\begin{aligned} \mathcal{H} = - \boldsymbol{\nabla}\boldsymbol{\cdot}\boldsymbol{\mathcal{F}}^{\mathrm{rad}}, \end{aligned}$$

as is often done in the simulations with the Rayleigh code (e.g. Featherstone and Hindman 2016; Bice and Toomre 2022). Yet another approach is to relax the thermodynamics toward a fixed reference state using a Newtonian cooling term as is done in the Eulag simulations (e.g. Ghizaru et al. 2010; Passos and Charbonneau 2014; Strugarek et al. 2018; Guerrero et al. 2019).

Typical numerical methods need to include some form of subgrid-scale (SGS) diffusion in the entropy equation to ensure numerical stability. In some methods, such as those used in the ASH, Rayleigh, and Pencil Code, this is explicitly included in a term that is proportional to the entropy gradient (e.g. Brun et al. 2004; Käpylä et al. 2013; Matilsky and Toomre 2020)

11 $$\begin{aligned} \boldsymbol{\mathcal{F}}^{\mathrm{SGS}} = - \chi_{\mathrm{SGS}}\rho T \boldsymbol {\nabla}s, \end{aligned}$$

where $\chi_{\mathrm{SGS}}$ is the SGS thermal diffusivity that is responsible for turbulent diffusion at unresolved scales. This definition implicitly assumes that $ds/dr < 0$, that is, that the turbulent diffusion is due to unresolved Schwarzschild unstable convection, and the SGS term contributes to a positive (outward) energy flux. Often it is advantageous to decouple the SGS diffusion from the mean stratification such that the SGS diffusion is applied not on the total entropy $s$ but, for example, to deviations from the spherically symmetric mean state $s' = s - \langle s \rangle$, where the overbar denotes suitable averaging, typically over the horizontal directions. This leads to a vanishing mean SGS flux, $\langle \boldsymbol{\mathcal{F}}^{\mathrm{SGS}} \rangle \approx0$. This is advantageous if part of the convection zone is weakly stably stratified, or a stably stratified radiative layer is taken into account below the convection zone (e.g. Brun et al. 2011; Käpylä et al. 2020). An alternative approach is to include SGS effects implicitly such that the effective diffusion at small scales is determined by the numerical scheme itself. This is done in, for example, the Eulag (e.g. Ghizaru et al. 2010) and R2D2 codes (e.g. Hotta et al. 2014).

In practice, all of the diffusion coefficients in the simulations are much larger than their counterparts in stars, e.g. such that $\nu\gg\nu_{\star}$, $\eta\gg\eta_{\star}$, where the subscript ⋆ refers to stellar values. Furthermore, the radiative diffusivity $\chi= K/(c_{\mathrm{P}}\rho)$ is also practically always much smaller than $\chi_{\mathrm{SGS}}$ (see Appendix A of Käpylä et al. 2017). Therefore all of the current simulations need to be understood as large-eddy simulations (LES), where the small unresolved scales typically modeled as enhanced diffusion coefficients. This is to be contrasted with direct numerical simulations (DNS) where $\nu= \nu_{\star}$, $\eta=\eta_{\star}$, and $\chi _{\mathrm {SGS}}= 0$. Furthermore, some models (e.g. Strugarek et al. 2016; Hotta et al. 2022) dispense with the explicit physical diffusion terms completely in order to minimize the diffusion on resolved scales while exerting adaptive diffusion at scales near the grid scale. These models are referred to as implicit LES (iLES) because the diffusion is built in into the numerical scheme without reference to physical diffusion terms.

### Dimensionless Parameters and Diagnostics

A number of non-dimensional parameters arise in the analysis of the MHD equations and which define the simulations. These include the Rayleigh number which describes the efficiency of convection

12 $$\begin{aligned} {\mathrm{Ra}}= \frac{gd^{4}}{\nu\chi} \left(- \frac{1}{c_{\mathrm{P}}} \frac{{\mathrm{d}}s}{{\mathrm{d}}r} \right), \end{aligned}$$

where $d$ is a length scale; typically taken to be the shell thickness, the thermal and magnetic Prandtl numbers describe the relative importance of various diffusion terms:

13 $$\begin{aligned} {\mathrm{Pr}}= \frac{\nu}{\chi},\ {\mathrm{Pr}}_{\mathrm{M}}= \frac{\nu}{\eta}, \end{aligned}$$

and the Taylor number

14 $$\begin{aligned} {\mathrm{Ta}}= \frac{4\Omega_{0} d^{4}}{\nu^{2}}, \end{aligned}$$

which measures the strength of rotation. The latter is related to the Ekman number via ${\mathrm{Ek}}= 2 {\mathrm{Ta}}^{-1/2}$. Most often the relevant thermal Prandtl number is based on the SGS diffusion

15 $$\begin{aligned} {\mathrm{Pr}}_{\mathrm{SGS}}= \frac{\nu}{\chi_{\mathrm{SGS}}}, \end{aligned}$$

because $\chi_{\mathrm{SGS}}\gg\chi$. For completeness, the viscosity $\nu$ used in simulations is also an effective or SGS viscosity because it is always much larger than the real physical value. However, it has the same functional form as the physical viscosity whereas a term corresponding to the SGS entropy diffusion does not appear in the original equations. Additionally the geometry and the resulting density stratification are input parameters of the models, along with the boundary conditions applied to the various quantities.

The most common diagnostic parameters used to describe the simulations include the Reynolds and Péclet numbers

16 $$\begin{aligned} {\mathrm{Re}}= \frac{u_{\mathrm{rms}}\ell}{\nu},\ {\mathrm {Re}}_{\mathrm{M}}= \frac{u_{\mathrm{rms}}\ell}{\eta}={\mathrm{Pr}}_{\mathrm {M}}{\mathrm{Re}},\ {\mathrm{Pe}}= \frac{u_{\mathrm{rms}}\ell}{\chi}=\Pr{\mathrm{Re}}, \end{aligned}$$

where $u_{\mathrm{rms}}$ is the rms-velocity and $\ell$ is a length scale, both of which are outcomes of the simulations. The latter is often defined as the integral scale; see e.g., Yadav et al. (2015b). The magnetic Reynolds number is of particular interest for dynamo simulations due to the bifurcations related to the excitation of large-scale and small-scale dynamos (SSD) (e.g. Brandenburg and Subramanian 2005; Rempel et al. 2023). The rotational effect on the flow is measured by the fluid Rossby (inverse Coriolis) number

17 $$\begin{aligned} {\mathrm {Ro}}_{\mathrm {f}}= \frac{u_{\mathrm{rms}}}{2\Omega_{0} \ell} \propto {\mathrm{Co}}^{-1}. \end{aligned}$$

An alternative way to define the Rossby number, which automatically takes the changing length scale into account is

18 $$\begin{aligned} {\mathrm{Ro}}_{\omega}= \frac{\omega_{\mathrm{rms}}}{2\Omega_{0}} \propto{ \mathrm{Co}}_{\omega}^{-1}, \end{aligned}$$

where $\omega_{\mathrm{rms}}$ is the rms-vorticity with $\boldsymbol{\omega}=\boldsymbol{\nabla}\times\boldsymbol{u}$. Order of magnitude estimates for some of these parameters in the deep parts of the solar convection zone and in a core convection zone of a $20~M_{\odot}$ O star in comparison to current simulations are listed in Table 1.

**Table 1:** Orders of magnitude of some dimensionless parameters in the main sequence phase of the Sun in the bulk of convection zone and in a core convection zone of a $20~M_{\odot}$ O9 star. Typical values from current global 3D simulations of stellar convection and dynamos are listed in the last column

Parameter	Sun ($M_{\odot}$)	O9 ($20~M_{\odot}$)	Simulations
Ra	10^20^	10^24^	10^9^
Pr	10^−6^	10^−5^	10^−1^…10
${\mathrm{Pr}}_{\mathrm{M}}$	10^−3^	10^3^	10^−1^…10
Re	10^13^	10^11^	10^4^
Pe	10^7^	10^6^	10^4^
${\mathrm{Re}}_{\mathrm{M}}$	10^10^	10^14^	10^4^
Δ*ρ*	10^6^	3	10^2^
Ro	0.1…1	1^1^	10^−2^…10^3^

Note: Solar values are from Ossendrijver (2003) and Schumacher and Sreenivasan (2020) whereas the values for the O9 star are from Jermyn et al. (2022). Δ*ρ* is the ratio of the fluid density between the bottom and top of the convection zone. We note that in Augustson et al. (2019) the Prandtl numbers for the O9 star are somewhat lower, i.e., Pr = 10^−6^ and ${\mathrm{Pr}}_{\mathrm{M}}= 10$; see their Fig. 2.

^1^Estimated using ${\mathrm{Ro}}= P_{\mathrm{rot}}/\tau_{\mathrm{conv}}$ where $\tau_{\mathrm{conv}}$ was taken from Fig. 74 of Jermyn et al. (2022) and the solar rotation period $P_{\odot}=27\text{ days}$ was used as a reference value for $P_{\mathrm{rot}}$.

Our discussion above has assumed that the *dimensional* MHD equations are being solved, in which case these non-dimensional parameters are diagnostic outputs of the simulations. An alternative approach, also employed by many authors (see, e.g. Gastine et al. 2016; Brown et al. 2020) is to non-dimensionalize the governing equations at the beginning; in this case the various non-dimensional parameters discussed here appear directly in the equations, and serve as input parameters that specify the problem. To illustrate the procedure, suppose we choose to measure lengths in units of a characteristic length $\ell_{\mathrm{c}}$, times in units of some time $\tau_{\mathrm {c}}$, velocities in units of $u_{\mathrm{c}}$, and temperatures in units of $T_{\mathrm {c}}$. That is, we assume $x = \ell_{\mathrm{c}} x_{\mathrm{nd}}$, $t = \tau_{\mathrm{c}} t_{\mathrm{nd}}$, and so forth, where the “$\mathrm{nd}$” subscript denotes non-dimensional variables. Then, to take a simple example, the dimensional Boussinesq momentum equation in the absence of rotation or magnetism in a plane layer,

19 $$ \frac{\partial\boldsymbol{u}}{\partial t} + \boldsymbol{u}\boldsymbol{\cdot} \boldsymbol{\nabla}\boldsymbol{u}= - \boldsymbol{\nabla}\varpi+ \nu\boldsymbol{\nabla}^{2} \boldsymbol{u}+ \alpha g T \hat{\boldsymbol{z}}, $$

where $\varpi\sim P/\rho$ is a reduced pressure and other symbols take their usual meanings, would be rewritten as

20 $$ \frac{u_{\mathrm{c}}}{\tau_{\mathrm{c}}} \frac{\partial\boldsymbol{u}_{\mathrm{nd}}}{\partial t_{\mathrm {nd}}} + \frac{u_{\mathrm{c}}^{2}}{\ell_{\mathrm{c}}} \boldsymbol {u}_{\mathrm{nd}} \boldsymbol{\cdot} \boldsymbol{\nabla}_{\mathrm{nd}} \boldsymbol{u}_{\mathrm{nd}} = - \frac{\varpi_{\mathrm{c}}}{\ell_{\mathrm{c}}} \boldsymbol{\nabla}_{\mathrm {nd}} \varpi_{ \mathrm{nd}} + \frac{u_{\mathrm{c}}}{\ell_{\mathrm{c}}^{2}} \nu \boldsymbol{\nabla}_{\mathrm{nd}}^{2} \boldsymbol{u}_{\mathrm{nd}} + \alpha g T_{c} T_{\mathrm{nd}} \hat {\boldsymbol{z}}, $$

where we have retained $\mathrm{nd}$ subscripts on all non-dimensional quantities (including the spatial and temporal derivatives), and where $\hat{\boldsymbol{z}}$ is the vertical unit vector. Many choices of the characteristic scales $\tau_{\mathrm{c}}$, $\ell_{\mathrm{c}}$, etc., are possible, and in general these will each yield slightly different forms of the non-dimensional equations. A common choice is to measure lengths in units of the convection zone thickness (${\equiv} L$), times in units of a thermal diffusion time across that length ($\tau_{\mathrm{c}} =L^{2}/\chi$), and to take $u_{\mathrm{c}} = L/\tau_{\mathrm{c}}$ for consistency; upon substitution (and simplification) we then find

21 $$ \frac{\partial\boldsymbol{u}_{\mathrm{nd}}}{\partial t_{\mathrm {nd}}} + \boldsymbol{u}_{\mathrm{nd}} \boldsymbol{\cdot}\boldsymbol{\nabla}_{\mathrm{nd}} \boldsymbol{u}_{\mathrm{nd}} = -\frac{1}{{\mathrm{Ma}}^{2}} \boldsymbol{\nabla}_{\mathrm{nd}} \varpi_{\mathrm{nd}} + {\mathrm{Pr}}\boldsymbol {\nabla}_{\mathrm{nd}}^{2} \boldsymbol{u}_{\mathrm{nd}} + {\mathrm{Ra}}{\mathrm{Pr}}T_{\mathrm {nd}} \hat{\boldsymbol{z}}, $$

now involving ${\mathrm{Ma}}^{2} = (P_{\mathrm{c}}/\rho_{\mathrm{c}})/u_{\mathrm {c}}^{2}$, ${\mathrm{Pr}}= \nu/\chi$, and ${\mathrm{Ra}}=g \alpha T_{\mathrm{c}} L^{3}/(\nu\chi)$ (versions of the Mach, Prandtl, and Rayleigh numbers) as *input* parameters.

An advantage of this approach is that it is easier to avoid inadvertently “running the same simulation twice” – that is, conducting calculations with different luminosities, rotation rates, etc., that are nonetheless functionally equivalent (because they have the same governing non-dimensional parameters). On the other hand, it is sometimes difficult to “re-dimensionalize” such calculations and so make contact with any given astrophysical object; for illustrations of the procedure and its ambiguities, see discussions in Jones et al. (2011) and Yadav et al. (2016). We generally adopt the “dimensional” view throughout the remainder of this review.

### Relevant Time and Length Scales in Stars

The structure of a star is determined by its mass $M$, luminosity $L$, chemical composition $\mu$, and rotation rate $\Omega_{0}$, the latter corresponding to its age. This information, along with material properties such as viscosity, opacity, equation of state, and nuclear energy production rate is in principle enough to construct a time-dependent model of the evolution of the star (e.g. Kippenhahn et al. 2012). However, in practice the evolution of main-sequence stars occurs over the nuclear timescale $\tau_{\mathrm{n}}$ which is of the order of $\tau_{\mathrm{n}}\approx10^{10}\text{ yr}$ for the Sun, which is much longer than what can be covered in any 3D dynamo simulation. Chemical evolution due to nuclear reactions occurs also in this timescale and therefore the solar and stellar dynamo simulations assume that the stellar structure is given and fixed in the course of the simulations (see, however Emeriau-Viard and Brun 2017). By the same token, the gravitational potential is assumed to be fixed and spherically symmetric. Rotational evolution of stars also happens on timescales of $10^{8}$ to $10^{9}$ years (e.g. Skumanich 1972; Barnes 2003; Gallet and Bouvier 2013) such that in 3D simulations the rotation rate of the star is assumed to be fixed. There is an ongoing debate based observational results suggesting magnetic braking slows down around the solar age which might be due to a transition to anti-solar differential rotation and a corresponding change in the dynamo (e.g. van Saders et al. 2016).

In general, the thermal evolution of the star in 3D simulations still occurs on a Kelvin–Helmholtz timescale

22 $$\begin{aligned} \tau_{\mathrm{KH}}= \frac{GM^{2}}{2RL}, \end{aligned}$$

where $G$ is the gravitational constant. The Kelvin-Helmholtz time for the solar convection zone is of the order of $10^{5}$ years which is still about two orders of magnitude longer than the longest global 3D simulations to date (Passos and Charbonneau 2014; Käpylä et al. 2016). Various ways to overcome or circumvent this issue are discussed below in Sect. 3.1. The final timescale related to stellar structure is the free-fall or acoustic timescale $\tau_{\mathrm{ac}} = \sqrt{R^{3}/GM}$, which is of the order of 30 minutes for the Sun. The limitations the timescales impose on simulations are discussed further in Sect. 3.2.

In terms of global dynamos the most important timescales are the rotation period $P_{\mathrm{rot}}$ and the convective turnover time,

23 $$\begin{aligned} \tau_{\mathrm{conv}}= \frac{\ell}{u_{\mathrm{conv}}}, \end{aligned}$$

where $\ell$ is the convective length scale and $u_{\mathrm{conv}}$ a suitably averaged convective velocity. The convective turnover time $\tau_{\mathrm{conv}}$ can be estimated from solar surface observation where granules overturn on a timescale of a few minutes. Knowledge of $\tau_{\mathrm{conv}}$ in deeper layers relies heavily on theoretical estimates, for example, from mixing length models (e.g. Böhm-Vitense 1958). These assume that the length scale is proportional to the pressure scale height. At the same time, convective velocities decrease such that $\tau_{\mathrm{conv}}$ is of the order of a month near the base of the solar convection zone (e.g. Stix 2002). Stellar observations indicate that dynamo efficiency of stars is related to the Rossby number (see, Sect. 5.2)

24 $$\begin{aligned} {\mathrm{Ro}}= \frac{P_{\mathrm{rot}}}{\tau_{\mathrm{conv}}}. \end{aligned}$$

The Rossby number is the only non-dimensional diagnostic that the simulations can capture relatively accurately; see Table 1 and Sect. 3.1. Equations (24) and (17) are related via ${\mathrm {Ro}}= 4\pi {\mathrm {Ro}}_{\mathrm {f}}$.

Another timescale that the simulations need to capture is the activity cycle period $\tau_{\mathrm{cyc}}$ which is 22 years for the Sun, and which varies between years to decades for stars other than the Sun (e.g. Baliunas et al. 1995; Hall et al. 2009; Olspert et al. 2018). It is, however, practically always necessary to run simulations considerably longer because establishing the global dynamo and reaching the final saturated dynamo mode takes typically significantly longer (e.g. Käpylä et al. 2013; Matilsky and Toomre 2020). Taking the thermal relaxation also into account, the integration times are typically at least an order of magnitude longer than the cycles established. The necessity to run such long times is one of the major limiting factors in the quest to reach astrophysically relevant parameter regimes.

In principle the relevant length scales vary between the depth of the convection zone $\Delta R$ (or the radius of the star $R$ for fully convective stars), and the Kolmogorov length scale $\ell_{\nu}$ where kinetic energy is dissipated into heat due to viscosity (or to the magnetic dissipation scale $\ell_{\eta}$ in cases where ${\mathrm{Pr}}_{\mathrm{M}}> 1$). According to the Kolmogorov turbulence phenomenology (e.g. Frisch 1995), $\ell_{\nu}$ can be estimated from the Reynolds numbers at the integral ($L$) and Kolmogorov scales,

25 $$\begin{aligned} \ell_{\nu}= L \left( \frac{{\mathrm{Re}}_{L}}{{\mathrm{Re}}_{\ell _{\nu}}} \right)^{-3/4}. \end{aligned}$$

For the Sun, $L=\Delta R = 2\cdot10^{8}\text{ m}$ and ${\mathrm{Re}}_{L} \gtrsim10^{12}$ (e.g. Ossendrijver 2003; Jermyn et al. 2022) and ${\mathrm{Re}}_{\ell_{\nu}} = 1$ by definition. These estimates yield an upper limit of order of magnitude $\ell_{\nu}\approx0.1\text{ m}$ near the base of the solar convection zone. More detailed calculations yield values between $0.01\text{ m}$ (Kupka and Muthsam 2017) and $0.06\text{ m}$ (Schumacher and Sreenivasan 2020). Furthermore, the dissipation scales of magnetic fields and temperature fluctuations can be estimated from $\ell_{\eta}= \ell_{\nu}{\mathrm{Pr}}_{\mathrm{M}}^{-3/4}$, and $\ell_{\chi}= \ell_{\nu}{\mathrm{Pr}}^{-3/4}$, respectively. Even though ${\mathrm{Pr}}_{\mathrm{M}}$, ${\mathrm{Pr}}\ll1$, these scales are also very small in comparison to the depth of the convection zone or the radius of the star. As will be discussed below, all of the scales where physical diffusion occurs are several orders of magnitude smaller than what can be achieved in any current or foreseeable simulations (see also Kupka and Muthsam 2017).

**Fig. 1:**
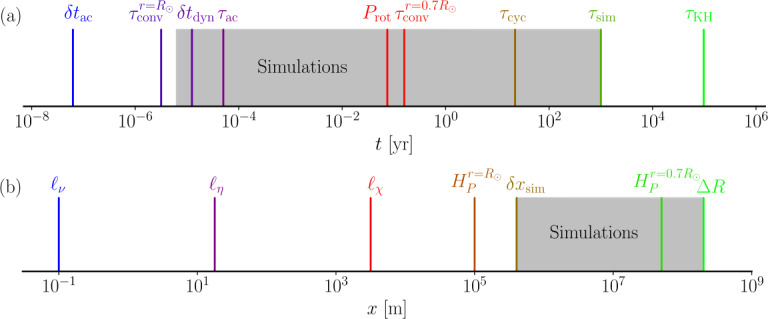
Orders of magnitudes of time (a) and length (b) scales in the deep solar convection zone. The gray shaded area indicates regions accessible to a typical current global simulation. $\delta_{\mathrm{ac}}$ and $\delta_{\mathrm{dyn}}$ are hypothetical acoustic and dynamical timesteps in a direct simulation of the solar convection zone; see Section 3.2

Another length scale that plays an important role is the pressure scale height $H_{\mathrm{P}}= - dr/d\ln p$ which is related to the vertical scale of convection cells. Near the surface $H_{\mathrm{P}}$ is of the order of $100\text{ km}$ in the photosphere of the Sun. On the other hand, at the base of the convection zone $H_{\mathrm{P}}\approx5\cdot10^{4}\text{ km}$. This reflects the fact that near the surface the pressure and density decrease very rapidly and that capturing both the deep and photospheric convection in a single model is therefore very challenging. In total the solar convection zone encompasses more than 20 pressure scale heights. This translates to a density difference of $10^{6}$ between the photosphere and the base of the convection zone. Estimates of the relevant temporal and length scales in the deep parts of the solar convection zone are summarized in Fig. 1; see also related discussion in Käpylä et al. (2013) and their Fig. 1.

## Numerical Approach to Stellar Dynamos

### Simulation Strategy

The main difficulty in solar and stellar dynamo simulations is that it is not feasible to match the dimensionless parameters with those of real stars as seen from the comparison of stellar and simulation parameters in Table 1. The only general exception to this is the Rossby number but even there we face the situation that only some of the convective scales in stars are strongly affected by rotation. For example, in the Sun the near-surface layers are practically unaffected by rotation whereas at the base of the convection zone the fluid Rossby number based on mixing length estimates of $\ell$ and $u_{\mathrm {rms}}$ is of the order of 0.1 (e.g. Ossendrijver 2003). This is to be contrasted with the Earth’s dynamo where the magnetic Reynolds number is ${\mathrm{Re}}_{\mathrm{M}}\approx 10^{3}$, which is within reach of current simulations (e.g. Aubert and Gillet 2021) and where all convective scales are strongly rotationally constrained (${\mathrm{Ro}}\approx10^{-6}$); see, e.g., Roberts and King (2013).

A path that stellar dynamo simulations often follow to approach physically relevant regimes is to assume a fixed convective Rossby number (Gilman 1977), given by

26 $$\begin{aligned} {\mathrm{Ro}}_{\mathrm{c}}= \left(\frac{{\mathrm{Ra}}}{{\mathrm {Pr}}{\mathrm{Ta}}}\right)^{1/2}. \end{aligned}$$

Here, the stellar luminosity fixes the level of driving through the Rayleigh number, and the stellar rotation rate is fixed by observations. Using typical estimates for ${\mathrm{Ra}}$, ${\mathrm{Pr}}$, and ${\mathrm{Ta}}$ for the Sun (Ossendrijver 2003, see also Table 1) we arrive at ${\mathrm{Ro}}_{\mathrm{c}}\approx0.1 \ldots1$. In simulations, the (SGS) Prandtl number ${\mathrm{Pr}}_{\mathrm{SGS}}=\nu/\chi_{\mathrm{SGS}}$ is often fixed and changing the diffusivities $\nu$ and $\chi_{\mathrm{SGS}}$ leads to ${\mathrm{Ra}}\propto\nu^{-2}$, ${\mathrm{Ta}}\propto\nu^{-2}$ and ${\mathrm{Ro}}_{\mathrm{c}}= \mathrm{{const.}}$ An obvious limitation is that the Prandtl number in simulations is typically close to unity whereas in stars ${\mathrm{Pr}}\ll1$ (e.g. Augustson et al. 2019; Schumacher and Sreenivasan 2020; Jermyn et al. 2022). A similar argument applies to ${\mathrm{Pr}}_{\mathrm{M}}$ in late-type stars whereas in the core convection zones of massive O and B stars ${\mathrm{Pr}}_{\mathrm{M}}\gg1$ (e.g. Augustson et al. 2016). Furthermore, in iLES models the values of the dimensionless parameters are typically unknown and not precisely controllable, although it is often possible to determine these *a posteriori* (e.g. Strugarek et al. 2016; Hotta et al. 2022). Nevertheless, the strategy in both LES and iLES models is to try to capture the stellar Rossby number with unrealistic Prandtl numbers and to resolve enough scales in an effort to reach sufficiently high ${\mathrm{Re}}$, ${\mathrm{Re}}_{\mathrm {M}}$, and ${\mathrm{Pe}}$ such that the large scale results are no longer affected. However, it is still questionable whether such a regime has been reached even in the highest resolution simulations to date (e.g. Hotta et al. 2022; Guerrero et al. 2022).

### Limitations of Current Numerical Simulations

The challenge of doing direct numerical simulations (DNS) of stars is illustrated by considering the solar convection zone where the fluid Reynolds number is of the order of at least $10^{12}$ (e.g. Ossendrijver 2003; Jermyn et al. 2022). With this estimate and Eq. (25), the ratio of the system scale $L$, here taken to be the depth of the solar convection zone or $200\text{ Mm}$, to the Kolmogorov scale $\ell_{\nu}$, is

27 $$\begin{aligned} \frac{L}{\ell_{\nu}} = \left( \frac{{\mathrm{Re}}_{L}}{{\mathrm{Re}}_{\ell_{\nu}}} \right)^{3/4}. \end{aligned}$$

With ${\mathrm{Re}}_{\ell_{\nu}}=1$, we obtain $L/\ell_{\nu }=10^{9}$. This ratio gives the order of magnitude of grid points that is required to capture all of the physically relevant scales in the solar convection zone. Thus a direct 3D simulation requires $10^{27}$ grid points. A somewhat lower, but still unattainable, number was reported in Chan and Sofia (1986).

Current state-of-the-art global simulations have of the order of $10^{10}$ grid points and are run on a few times $10^{4}$ CPU cores. Assuming ideal weak scaling, where the computation time remains constant when the number of CPUs is increased proportional to the grid size, a DNS of the solar convection zone requires $10^{21}$ CPU cores. Using a current 96-core AMD Epyc™ 9654 CPU with 360 W thermal design power as a reference,^1^ gives a total power consumption of $3.8\cdot10^{21}\text{ W}$, corresponding roughly to a M9V main-sequence red dwarf. It is clear that such power is neither available nor meaningful to be spent. Although reaching an asymptotic regime where the large-scale dynamics are unaffected by the addition of further small scales is very likely possible at a significantly lower resolution, it is clear that even the highest resolution current simulations are not there yet (e.g. Käpylä et al. 2017; Hotta et al. 2022).

Furthermore, the timestep in such hypothetical DNS of the solar convection zone is of the order of $\delta t \approx\ell_{\nu}/{\mathrm{max}}(c_{\mathrm {sig}}^{\mathrm{max}})$, where $c_{\mathrm{sig}}^{\mathrm{max}}$ is the maximum signal propagation speed. In anelastic models this is set by the maximum flow velocity which is of the order of 1 km s^−1^, whereas in the fully compressible case this is the sound speed $c_{\mathrm{s}}$, which at the base of the convection zone is around 200 km s^−1^. This gives $\delta t = \delta t_{\mathrm{dyn}} \approx2 \times 10^{-4}\text{ s}$ for anelastic and $\delta t = \delta t_{\mathrm{ac}} \approx10^{-6}\text{ s}$ for fully compressible models. In practice, the resolution is much lower and corresponding estimates for a high-resolution global simulation with 500 uniformly spaced grid points in radius gives $\delta t_{\mathrm{ac}} \approx2\text{ s}$ and $\delta t_{\mathrm{dyn}} \approx10\text{ minutes}$. The latter is still longer than the convective turnover time near the surface of the Sun, where $\tau_{\mathrm{conv}}^{r=R_{\odot}} \approx1\text{ minute}$. The surface of the Sun is extremely challenging to be taken into account in a global model due to a combination of very small length scales and short time scales and the Mach number approaching unity. Therefore a full Sun simulation requires a numerical scheme capable of dealing with practically all Mach numbers and multiscale convection. Furthermore, the boundary region where radiative cooling takes place near the surface is extremely thin, around 10 km, in comparison to the depth of the convection zone (Kupka and Muthsam 2017). Typically simulations either do not reach all the way to the photosphere, or consider a shell reaching to $R=R_{\odot}$ but with a much lower density stratification than in the Sun, and the boundary layer near the surface is made artificially thicker to resolve it numerically.

Another constraint arises due to a widening discrepancy of the timescales involved when resolution is increased: as was discussed earlier, a simulation of the Sun needs to cover at least a solar cycle or preferably several cycles to be considered viable such that the simulated time $\tau_{\mathrm{sim}} \gtrsim\tau_{\mathrm{cyc}}$. For the sake of argument, an acceptable maximum wall-clock time that a simulation is permitted to run to is taken to be a year. This requires that the star in the simulation has to evolve at least 22 times faster than in real time. However, when the grid resolution is increased, the timestep in explicit time-stepping methods decreases in proportion with the grid spacing $\delta x$, and the computational cost of simulation increases with $\delta x^{4}$. This corresponds to ${\mathrm {Re}}^{3}$, making it very difficult to reach high ${\mathrm {Re}}$. Even if the numerical scheme has ideal weak scaling, the time to solution doubles every time the resolution is doubled, which typically cannot be avoided. This poses stringent constraints on either the length, or the grid resolution, of simulations targeting global stellar dynamos. The timestep constraints can, to a certain degree, be alleviated by the use of implicit time stepping methods (e.g. Viallet et al. 2011) or by the use of local subgrids and timesteps (e.g. Popovas et al. 2022).

A further complication arises due to the thermal relaxation. In anelastic models, where the real stellar luminosity is often used, the Kelvin-Helmholtz time is much longer than the integration times of simulations. However, this is a worst-case scenario because deep stellar convection zones are nearly adiabatic which is exploited in the simulation setups. Thermal relaxation can still take a prohibitively long time if a stably stratified radiative layer is retained below the convection zone. This issue is sometimes alleviated by adjusting the radiative conductivity in the overshoot layer below the convection zone (e.g. Brun et al. 2017). However, this can lead to over- or underestimation of convective overshooting depending when and how such adjustments are made (Käpylä 2019). Another possibility is to adjust the thermodynamic state and fluctuations recursively toward an equilibrium solution (Anders et al. 2018, 2020), although this method has yet to gain widespread adoption in compressible or global 3D simulations.

In fully compressible simulations the timestep would be very short because it is determined by the sound speed at the base of the convection zone. This has been circumvented by the reduced sound speed technique (RSST) where the sound speed is artificially lowered such that the timestep issue is alleviated (e.g. Hotta et al. 2014). Another approach is the enhanced luminosity method (ELM) where a luminosity that is much higher than in real stars is used (e.g. Käpylä et al. 2013, 2020), leading to a higher Mach number and therefore a diminished gap between the acoustic and dynamical timescales, as well as a correspondingly shorter Kelvin-Helmholtz time. Given that the luminosity enhancement is sufficiently large, it is possible to resolve the Kelvin-Helmholtz timescale using fully compressible MHD equations (e.g. Käpylä 2023). The cost of this method is that in addition to higher flow velocities, also the thermodynamic fluctuations are enhanced, and a direct comparison with observations requires the use of scaling relations. Furthermore, to achieve the same Rossby number as in a real star with realistic luminosity, the rotation rate has to be increased in proportion to the Mach number, which would lead to unrealistically large centrifugal force (e.g. Navarrete et al. 2022).

### Numerical Methods and Codes

There are a variety of codes solving the MHD equations in spherical shells and targeting solar and stellar global dynamos. The first and still popular approach is to adopt the anelastic approximation where the sound waves are filtered out by neglecting the time derivative in continuity equation. Then it is convenient to use spherical harmonics to solve for the horizontal dynamics whereas the vertical discretisation is often done with finite differences or Chebychev polynomials. Codes using this approach include ASH (e.g. Clune et al. 1999; Brun et al. 2004; Jones et al. 2011; Brun et al. 2022), Rayleigh (Featherstone et al. 2022), and MagIC (e.g. Gastine and Wicht 2012). Eulag is another anelastic code but instead of spherical harmonics, it relies on a second-order accurate multidimensional positive-definite advection transport algorithm (MPDATA) and implicit time stepping (e.g. Smolarkiewicz and Charbonneau 2013).

Another popular technique is to use the fully compressible formulation and using some flavour of finite difference methods. This typically leads to coordinate singularities at the axis and at the centre of the star, which are circumvented by either omitting regions near the axis (spherical wedge, cf. Käpylä et al. 2012; Mabuchi et al. 2015), using partially overlapping grids (yin-yang grid cf. Hotta et al. 2015), or embedding a spherical star into a Cartesian cube (star-in-a-box model, cf. Dobler et al. 2006; Käpylä 2021). The Mach number issue of fully compressible simulations is dealt with by RSST and ELM methods that were discussed above. Codes using fully compressible formulation include the Pencil Code (Pencil Code Collaboration et al. 2021), R2D2 (Hotta et al. 2015), and the code used in Mabuchi et al. (2015). Further methods include the Dedalus framework which uses spectral methods and is capable of solving incompressible, anelastic, and fully compressible equations in varying geometries (Brown et al. 2020; Burns et al. 2020; Anders et al. 2022a), and the Dispatch framework where various solvers for compressible flows are possible and which uses local subdomains and timesteps (Nordlund et al. 2018; Popovas et al. 2022).

## Relevant Solar and Stellar Observations

The dynamo simulations discussed in this review aim, ultimately, to capture the flows and magnetism occurring in real stars. Here, we briefly describe some of the most pertinent observational constraints on these processes, which also serve to motivate and guide our work. Perhaps the most obvious characteristics that any dynamo simulation would hope to match are the Sun’s observed differential rotation and its periodic cycle of magnetic activity.

**Fig. 2:**
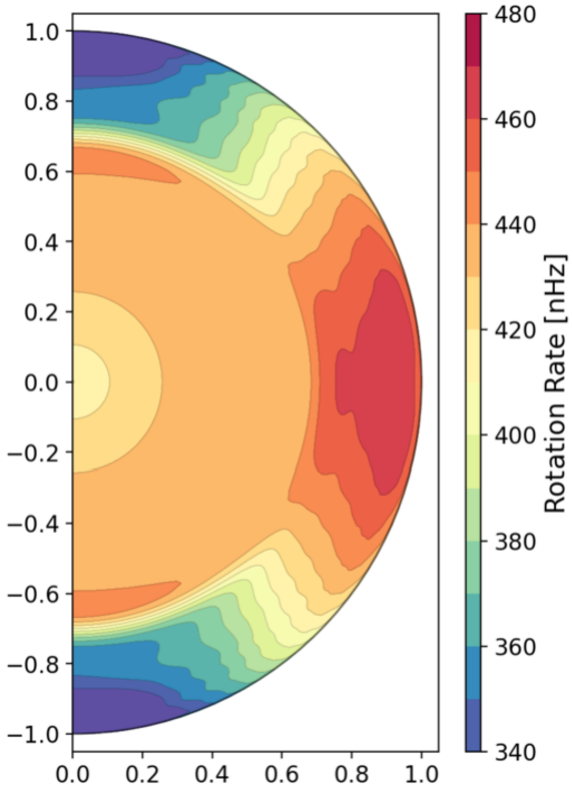
Solar differential rotation profile $\Omega/2\pi$ as a function of radius and latitude as inverted from helioseismology using the first 6 years of HMI 72-day analysis. Reddish colors indicating fast rotation and blueish tones slow rotation (adapted from Larson and Schou (2018) using the data archived at http://jsoc.stanford.edu/HMI/Global_products.html)

In Fig. 2, we sample the solar interior rotation profile as revealed by helioseismology (e.g. Schou et al. 1998). The Sun’s surface differential rotation – with a fast equator and a slow pole – imprints through the convection zone, with nearly solid-body rotation in the portions of the radiative zone below that are accessible to these global-scale inversions. There are two prominent shear layers – the tachocline near the base of the convection zone and the near-surface shear layer (NSSL) in the upper portions of the convection zone. The apparent width of the tachocline in this representation reflects the width of the inversion kernels used; its true width is thought to be narrower. Note, too, that although the shear within the convection zone has not spread through the radiative interior, the *average* rotation rate of the interior is commensurate with the average rate of the envelope; since the Sun is continuously losing angular momentum via its magnetized wind, and so spun more rapidly in the past, this observation implies some level of coupling between the two regions (Gilman et al. 1989; MacGregor and Brenner 1991; Spiegel and Zahn 1992; Gough and McIntyre 1998; Brun et al. 2011; Matt et al. 2015).

Ideally, a simulation would self-consistently capture at least a few key attributes of this profile: e.g., the overall pole-to-equator shear within the convection zone; the fact that isocontours of $\Omega$ are more nearly aligned with radius than they are with the rotation axis, in evident tension with the Taylor-Proudman theorem (e.g. Miesch et al. 2006); and the existence and properties of both the NSSL and the tachocline. In practice each of these remain a challenge, though as discussed later in this review (Sect. 5.1), the latest 3D MHD global simulations of solar interior dynamics and angular momentum transport do manage to capture many of these elements without undue tinkering.

**Fig. 3:**
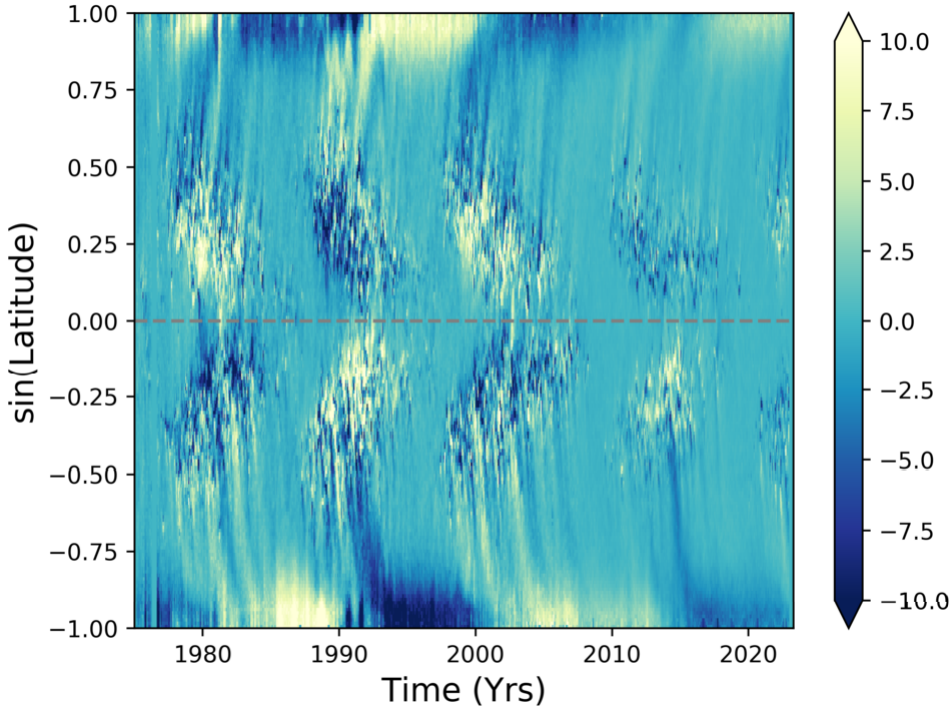
Solar butterfly diagram of the line of sight surface magnetic field up to Carrington rotation 2265 (Brun et al. 2020)

The most striking observational constraints on the magnetism involve its systematic evolution with space and time, as sampled in the famous “butterfly diagram.” An example is provided in Fig. 3, which shows the longitudinally-averaged line of sight component of the magnetic field for every Carrington rotation since 1975 (based on Wilcox, GONG and Solis synoptic map data; Brun et al. 2020). Strong fields emerge at mid-latitudes and then, over the course of roughly 11 years, appear progressively nearer the equator; the polarity of these emergent fields is the same for most of the low-latitude events in the Northern hemisphere, and opposite to that in the Southern; the overall polarity of the field flips at the end of each 11-year period. There is also a prominent polar branch of activity, which is at its strongest when the equatorial branch is at its ebb; the polarity of this polar branch matches that of the *following* equatorward branch. The polarity of the poloidal field thus reverses when active region emergence is at its maximum. The overall number of sunspots visible over the solar surface rises and falls over the course of the cycle, in line with the surface distribution of the strongest fields.

**Fig. 4:**
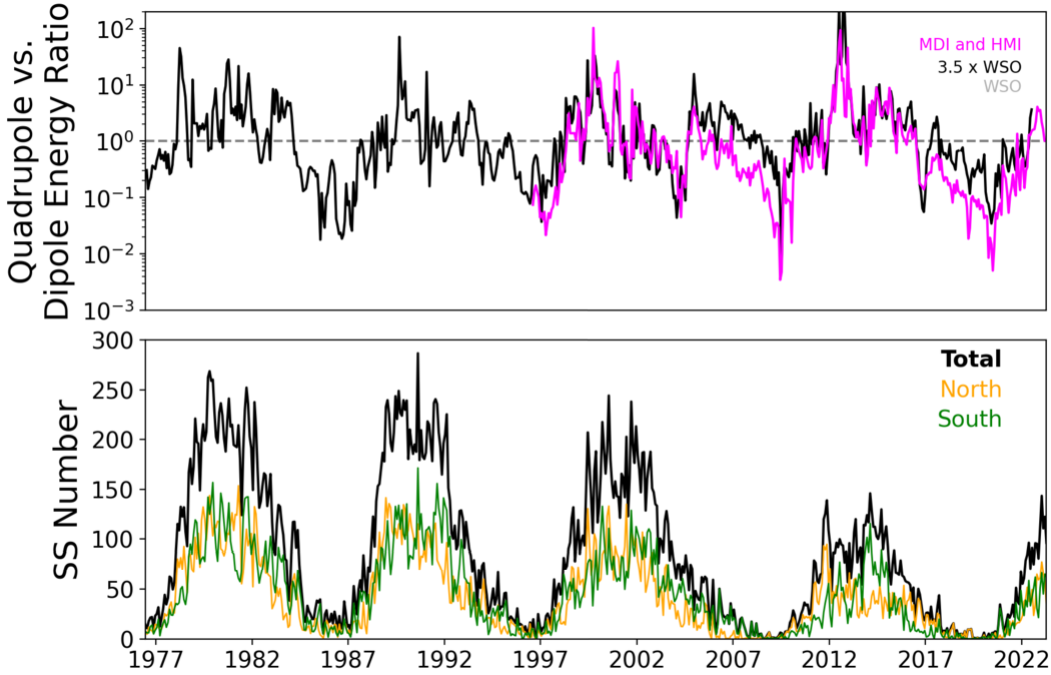
Top: Ratio of the solar magnetic dipole and quadrupole energies over the last few cycles (MDI and HMI Data). We note that the quadrupole modes dominate during the maximum cycle phase and this has already started for cycle 25 in 2022. This strong quadrupolar component also explains the time lag between the northern and southern hemispheres that can reach up to 18 months. Bottom: Sunspot numbers from all data and separately from northern and southern hemispheres. Adapted from Brun et al. (2020)

On the whole, the Sun’s ordered field exhibits dipole parity throughout most of the cycle (it is antisymmetric about the equator), but there are periods near cycle maximum during which the parity is mostly quadrupolar. The relative amplitudes of the dipolar and quadrupolar modes are shown in Fig. 4 over the past few cycles (Brun et al. 2020). These relations constitute another powerful constraint on dynamo models. For example, there is evidence that around the pronounced period of low surface activity known as the Maunder minimum, the Sun’s observed surface activity was predominantly confined to one hemisphere, indicating different parity relations; the implications of this finding for dynamos generally (and grand minima in particular) have been considered by, e.g., Sokoloff and Nesme-Ribes (1994) and in many subsequent papers.

Very recently, new constraints have begun to emerge from the study of inertial and Rossby wave modes that propagate within the convection zone. These toroidal modes have recently been observed in helioseismic maps of near-surface horizontal flows obtained by HMI aboard SDO (Gizon et al. 2021); see also Hanson et al. (2022) for another recent detection of solar inertial modes. Though modeling of these modes is still in its infancy (Bekki et al. 2022; Triana et al. 2022) they appear to hold great promise for constraining aspects of the convection that would be difficult or impossible to estimate by other means. As a first application, Gizon et al. (2021) illustrate that these modes constrain the superadiabaticity and turbulent diffusivity of the deep solar convection zone.

**Fig. 5:**
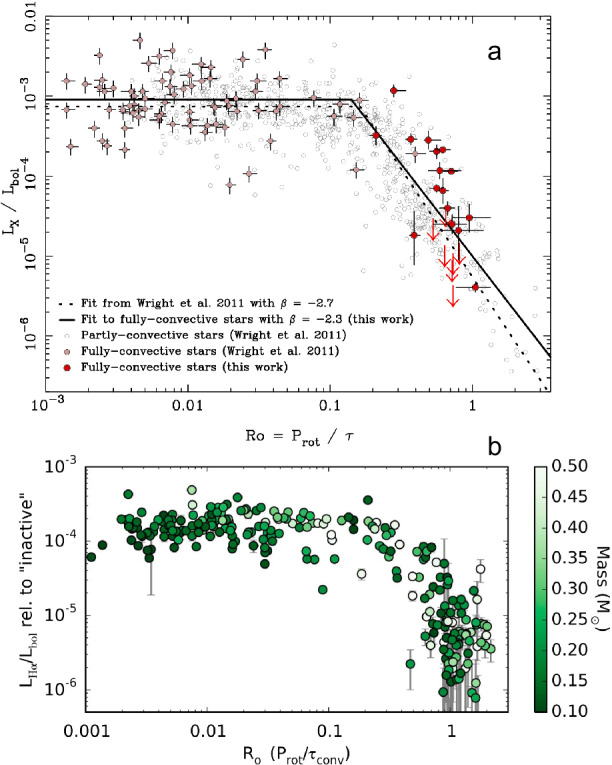
Relations between rotation rate (quantified by the Rossby number) and proxies of magnetic activity. ($a$) X-ray emission (normalized to the bolometric luminosity) for a sample of both fully and partially convective stars (Wright et al. 2018); ($b$) a measure of chromospheric H$\alpha$ emission, normalised to the bolometric luminosity and relative to that in inactive stars, in a sample of M dwarfs (Newton et al. 2017) (©AAS. Reproduced with permission)

**Fig. 6:**
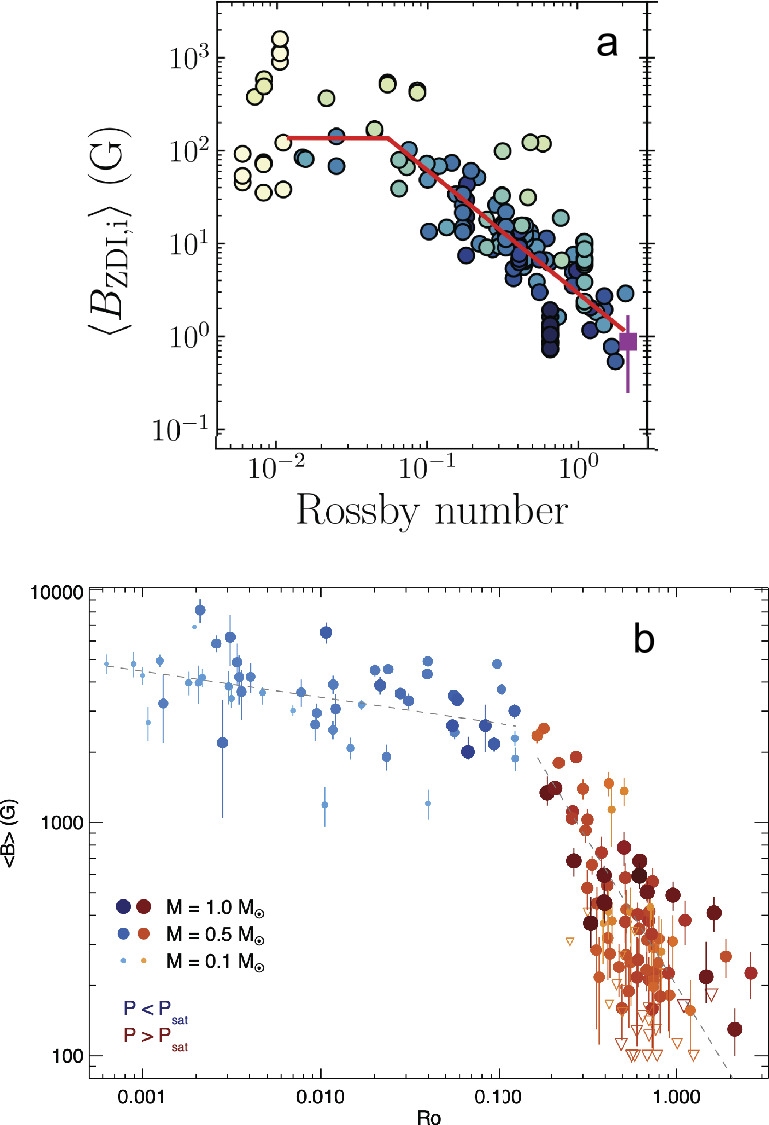
Similar to Fig. 5 but showing measures of magnetic field as a function of ${\mathrm {Ro}}$. ($a$) Zeeman Doppler imaging estimates of dipole component of surface magnetic field (See et al. 2019) (©AAS. Reproduced with permission). ($b$) Average surface magnetic field as measured by Zeeman broadening of spectral lines (Reiners et al. 2022) (©ESO. Reproduced with permission)

Finally, we turn briefly to observations of other stars. Many aspects of observational stellar magnetism are treated in other reviews (e.g. Reiners 2012; Brun and Browning 2017; Jeffers et al. 2023) so we note only a few key constraints that must eventually be matched by simulations. The most celebrated of such constraint is the “rotation-activity correlation,” sampled in Figs. 5 and 6. In stars with convective envelopes, surface measurements of magnetic activity first increase with rotation rate, then plateau (“saturate”) at a certain point. Here we show examples in which activity is measured by coronal emission (Wright et al. 2018), here including both fully convective and partially-convective stars in Fig. 5(a); by chromospheric H$\alpha$ emission (as a fraction of the bolometric luminosity) in a large sample of M dwarfs (Newton et al. 2017) in Fig. 5(b); via Zeeman Doppler Imaging (See et al. 2019), here providing an estimate of the large-scale dipole field observed at the surface (Fig. 6(a)); and via measurements of the surface magnetic field strength as revealed by the Zeeman broadening of spectral lines (Reiners et al. 2022); see Fig. 6(b). Typically in these studies the influence of rotation is characterized via a simple estimate of the Rossby number where the convective turnover time is typically based on simple empirical relations that work well for main-sequence stars (e.g. Noyes et al. 1984). When viewed in this way, many different types of stars – including those with and without a stable radiative region – appear to exhibit the same basic relationships. Comparisons of young and evolved main-sequence stars also suggest a similar level of activity as function of ${\mathrm{Ro}}$ in terms of Ca II H&K emission, provided that the convective turnover time is an outcome of 1D stellar models (Lehtinen et al. 2020).

The rotational velocity of a solar-like star changes systematically over time, as it loses angular momentum through a magnetised wind, so over the course of its life it will trace a variety of positions on this rotation-activity correlation. Indeed, measurements of rotation rate are used in “gyrochronology” as proxies for age, because many stars are found to exhibit a common, tight relation between spin rate and time – the so-called Skumanich law $\Omega\propto t^{-1/2}$; see discussions in Skumanich (1972), Soderblom (1983), and Barnes (2003). Lately there have been some indications that this relationship may break down at late ages (e.g. van Saders et al. 2016), which may provide additional constraints on the dynamo for old stars (e.g. Metcalfe and van Saders 2017). In a related vein, there is some evidence for enhanced stellar activity in a subset of slowly-rotating stars, which some authors have suggested may be linked to the presence of strong anti-solar differential rotation (Brandenburg and Giampapa 2018). Global dynamo simulations seem to capture this effect; see Karak et al. (2015), Warnecke and Käpylä (2020), Brun et al. (2022), and Noraz et al. (2023).

There has been a rising interest in efforts to detect anti-solar differential rotation observationally partly due to it being a robustly appearing feature in simulations. This is a challenging task because anti-solar differential rotation is expected to occur in slowly rotating stars where even the detection of the rotation period is difficult. Nevertheless, detections have been made from giant stars using Doppler imaging (e.g. Weber et al. 2005) by tracking the drift of spots on the surfaces of stars. Furthermore, Reinhold and Arlt (2015) developed a method to distinguish between solar-like and anti-solar differential rotation from long-term photometry. In their sample of 50 Kepler stars, they found that 10-20 per cent of these stars are likely to have anti-solar differential rotation. Noraz et al. (2022) identified 22 solar-type Kepler stars as anti-solar differential rotation candidates by inferring the Rossby numbers these stars are likely to have based on their rotation rate, interior structure, and metallicity. Finally, Benomar et al. (2018) used asteroseismology to estimate the latitudinal differential rotation in a sample of 40 Kepler stars. While none of the stars in their sample unambiguously showed anti-solar differential rotation, the methodology could in principle be used to detect it.

Together, these observations of the Sun and other stars constitute powerful constraints that models would hope to satisfy. In the following sections, we will examine the extent to which they actually do so.

## Simulations of Solar and Stellar Dynamos

### Convection and Dynamo in the Current Sun

In addition to the fundamental numerical restrictions discussed above, simulations of the current Sun are challenging due to the possible proximity of the transition from solar-like to anti-solar differential rotation. This transition occurs around ${\mathrm{Ro}}\approx1$ (e.g. Käpylä et al. 2011a; Gastine et al. 2014; Brun et al. 2017) and current simulations of the Sun appear to lie close to this in parameter space. Simulations with the nominal solar luminosity and rotation rate land predominantly in the anti-solar regime (e.g. Käpylä et al. 2014), which is one of the manifestations of the convective conundrum (O’Mara et al. 2016), or the lower than expected velocity amplitudes and Rossby number in the Sun in comparison to theoretical estimates and simulations, which is discussed in more detail elsewhere (e.g. Hanasoge et al. 2016). Therefore simulations targeting the Sun often resort to lowering the Rossby number artificially to obtain a solar-like differential rotation profile. This is most often done by suppressing the convective velocity by enhancing radiative diffusion (e.g. Käpylä et al. 2014; Fan and Fang 2014; Hotta et al. 2016; Noraz 2022), lowering the luminosity (e.g. Hotta et al. 2015; Guerrero et al. 2022), or by increasing the rotation rate.

The importance of reproducing the solar interior rotation lies in the fact that the dynamo process crucially relies on flows of various scales to maintain the observed magnetic field. At the very least, the large-scale flows are employed by all of the currently predominant solar dynamo models (Charbonneau 2020). Furthermore, it is also likely that turbulent effects, such as an $\alpha$ effect due to helical convection-driven turbulence, are important in the dynamo process. This highlights the importance of accurate modelling of convection, essentially necessitating that the solar velocity field has to be sufficiently well reproduced first before one should expect success in reproducing the dynamo. An important step in this is the identification of the relevant force balances that need to be reproduced in simulations. Following such an approach, a path in parameter space may be found that leads to solar-like results with feasible numerical cost. Such “path approach” is quite commonly used now in geodynamo modelling (Aubert et al. 2017; Aubert and Gillet 2021).

The convective conundrum is arguably the greatest obstacle in achieving the goal of simulating the solar dynamo successfully. Several ideas have recently been invoked to alleviate the discrepancy between models and reality. One of these ideas is that this is a manifestation of rotationally constrained convection in the interior of the Sun. In this scenario the maximum scale of convection in the deep parts of the solar convection zone is not giant cells, as is expected from mixing length models, but it matches instead that of the supergranulation, which is also detected from surface observations (Featherstone and Hindman 2016; Vasil et al. 2021). However, some such studies assume from the outset that convection is rotationally constrained in the Sun although it is unclear if this really is the case. For example, Featherstone and Hindman (2016) match a rapidly rotating simulation with the Sun based on the fact that the velocity power spectrum peaks at supergranular scale. No further independent check, for example by means of a Rossby number depending only on stellar parameters and not on estimates of convective velocities or scales (e.g. Käpylä 2023), is made that the simulation matches the relevant solar parameters. There is currently no simulation of solar convection that unambiguosly reaches a rotationally constrained state. Another caveat is that while simulations of rotationally constrained convection do produce smaller convective scales that become smaller as rotation becomes more rapid (e.g. Viviani et al. 2018), the main contribution to differential rotation in such models is still due to giant cell convection (e.g. Käpylä 2023) or thermal Rossby waves that have not yet been detected in the Sun.

Another idea that has gained popularity recently is that the deep parts of the solar convection zone can be weakly stably stratified. This is thought to result from strong driving of convection in the near-surface layers, whence plumes of cool low entropy material plough through the whole convection zone and deep into the stably stratified layers below. Such idea of *cool entropy rain* was put forward by Spruit (1997) and later incorporated into a modified mixing length model by Brandenburg (2016). In the extreme versions of these models only a very thin layer (down to a few Mm) near the surface of the convection zone is Schwarzschild unstable and the rest of the convection zone is weakly subadiabatic and mixed by the entropy rain. Such effects were explored in 3D simulations by Nelson et al. (2018) by means of a boundary condition consisting of localised cooling patches. Although non-rotating simulations often find relatively deep subadiabatic layers (e.g. Roxburgh and Simmons 1993; Tremblay et al. 2015; Hotta 2017; Käpylä et al. 2017), their effect in global simulations appears to be weak (Käpylä et al. 2019; Viviani and Käpylä 2021). This could also be due to the modest resolutions and supercriticality of convection in those studies.

Furthermore, the influence of the thermal Prandtl number has also recently been studied. In particular, several studies have concentrated on cases where the effective Prandtl number is greater than unity (e.g. O’Mara et al. 2016; Bekki et al. 2017; Karak et al. 2018). This was motivated by the observation that the overall velocities are decreased in high-${\mathrm{Pr}}$ convection. However, this also coincides with more effective downward flux of angular momentum, exacerbating the problems related to anti-solar differential rotation (Karak et al. 2018). Another recent study (Käpylä 2023) confirmed these ideas and showed that the Prandtl number dependence is relevant in the regime ${\mathrm{Pr}}\gtrsim1$, whereas for ${\mathrm{Pr}}\lesssim1$, the parameter regime relevant for the Sun, no statistically significant dependence was detected.

Finally, the role of magnetism in shaping the solar rotation profile is also a viable option to explain the convective conundrum. Whereas early forays into this field yielded somewhat contradictory results with some studies finding essentially no dependence on magnetic fields (Karak et al. 2015), others reported a flip from anti-solar to solar-like differential rotation (Fan and Fang 2014; Simitev et al. 2015). All of these simulations were made at relatively modest magnetic Reynolds numbers, and it is likely that a SSD was not excited in these models. The recent high-resolution implicit large-eddy simulations (iLES) (Hotta and Kusano 2021; Hotta et al. 2022), have reached a regime where small-scale magnetic fields are generated throughout the convection zone and turn a hydrodynamically anti-solar run to a solar-like solution at the highest resolution. Somewhat worryingly, these simulations have yet to show convergence as a function of resolution such that the flows at large scales changes significantly even between the two highest resolutions. Recently, Käpylä (2023) reported that it is easier to excite solar-like differential rotation for higher ${\mathrm{Re}}_{\mathrm {M}}$ from simulations with explicit diffusivities where ${\mathrm{Re}}_{\mathrm{M}}$ exceeded the threshold for SSD. However, this effect is much less drastic than in the iLES simulations.

The radial shear in the solar convection zone occurs predominantly in the boundary layers which are difficult to incorporate in global simulations. The near-surface shear layer is thought to be generated in the weakly rotationally constrained small-scale convection in the outermost parts of the solar convection zone (e.g. Kitchatinov 2016) or due to gyroscopic pumping effects (e.g. Miesch and Hindman 2011). Capturing this in global simulations is challenging because a very high resolution is required to capture the near-surface small-scale convection resulting from a steep decrease of fluid density. First such simulations were presented by Hotta et al. (2015), who were able to capture some aspects of the NSSL. However, these simulations were hydrodynamic and no corresponding dynamo solutions have been presented so far. The NSSL has also been suggested to shape the global solar dynamo (Brandenburg 2005), but no direct evidence supporting or refuting this theory is currently available.

The other boundary layer at the interface of the convective and radiative layers, the tachocline, is perhaps even more challenging to capture in simulations. The main challenge is that the solar tachocline is very thin (certainly less than five per cent of solar radius, but likely much less), and it has been confined now for five billion years. Estimates of radiative spreading for the Sun suggest that the tachocline should be much thicker at the current age of the Sun so there has to be a mechanims preventing this. Several magnetic scenarios have been invoked to explain this, including a dipolar fossil field in the radiative core or a cyclic dynamo in the convection zone. Some current simulations do exhibit tachocline-like features, but they are spreading into the radiative core at rates that are much higher than expected for the Sun (e.g. Brun et al. 2011, 2017). Furthermore, iLES models also produce tachoclines at relatively low resolutions, although their confinement mechanism is yet to be understood (Guerrero et al. 2013, 2016, 2022). In all of the aforementioned simulations the diffusivities in the radiative interior were either explicitly or implicitly greatly reduced. In apparent contradiction to these models, Matilsky et al. (2022) were able to obtain a relatively thin tachocline and essentially rigidly rotating radiative core in a simulation where the diffusivities were not decreased but which housed a cycling non-axisymmetric dynamo in the convection zone. Furthermore, this model has also strong horizontal flows in the radiative interior. The actual process of tachocline confinement is still unclear in this case, although it does share some characteristics with the cyclic dynamo confinement process suggested by Forgács-dajka and Petrovay (2001); see also Barnabé et al. (2017).

**Fig. 7:**
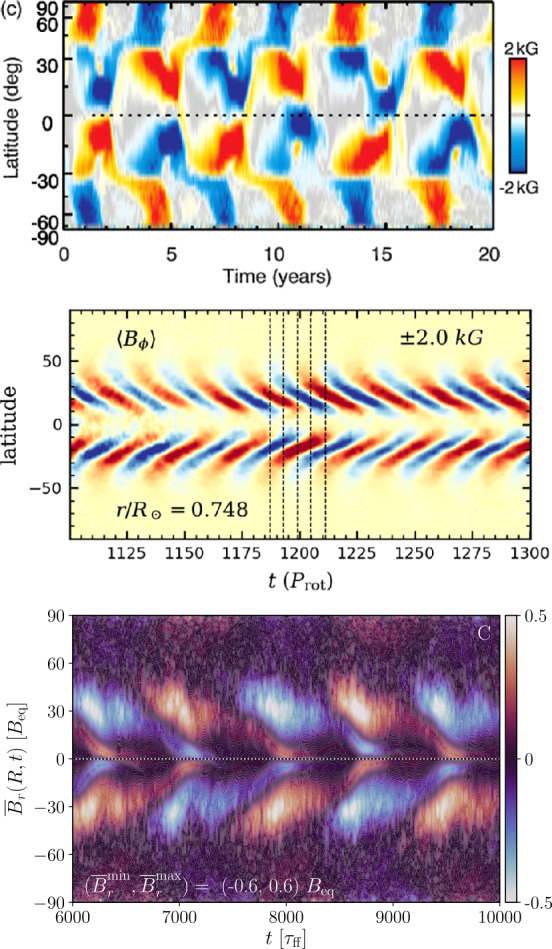
Butterfly diagrams from Augustson et al. (2015) (top), Matilsky and Toomre (2020) (middle; ©AAS. Reproduced with permission) and Käpylä (2022) (bottom)

Given the difficulties in reproducing the solar flows, it is then hardly surprising that dynamo simulations have had a hard time reproducing the solar large-scale magnetism. The most severe issue is the difficulty in obtaining solar-like equatorward migration of activity belts in simulations with solar-like differential rotation. Nevertheless, several simulations have appeared showing equatorward migration and which capture many aspects of solar observations. For example, Käpylä et al. (2012) reported equatorward migration from spherical wedge simulations that were later shown to be in accordance with a Parker–Yoshimura dynamo wave resulting from a mid-latitude minimum of angular velocity which is not present in the Sun (Warnecke et al. 2014, 2018). A similar mid-latitude dip is seen also in the equatorward migrating solutions of Augustson et al. (2015). Further examples of equatorward propagating solutions have been reported by Duarte et al. (2016), Matilsky and Toomre (2020), Strugarek et al. (2017, 2018), and Brun et al. (2022); see also Fig. 7. The latter authors argued that a non-linear interplay between the magnetic fields and differential rotation can lead to solar-like long period cyclic dynamos. Another recent example shows equatorward migration near the surface but poleward migration at depth in a star-in-a-box model (Käpylä 2022), where a spherical star is embedded into a Cartesian cube. In such models the boundary of the star is immersed into the domain and, in theory, allows for a more realistic magnetic boundary condition. This was shown to be important in that if the exterior was made a poor conductor, the global dynamo solution changed from oscillatory to quasi-static. This confirms earlier results of Warnecke et al. (2013, 2016), where the influence of a simplified coronal layer as upper boundary on the flow and magnetic field evolution was studied.

These simulation results and their interpretation add to the ongoing debate regarding the location and dominant physical mechanisms of the solar global dynamo. The current state of affairs is particularly clearly manifested by the wide variety of mean-field models that have been put forward to explain the solar cycle. A popular class of models include flux-transport (e.g. Dikpati and Charbonneau 1999) and Babcock-Leighton (e.g. Cameron and Schüssler 2017) dynamos where a minimal set of physically plausible ingredients, such as differential rotation and meridional circulation and the decay of active regions near the solar surface are taken into account. These models typically rely on buoyantly rising flux ropes (e.g. Caligari et al. 1995) that are the result of strong shear in the tachocline, and the turbulent flows in the convection zone play only the role of turbulent diffusion. On the other hand, distributed turbulent dynamos take into acount a variety of effects that arise in mean-field electrodynamics and assume magnetic field generation throughout the convection zone (e.g. Brandenburg et al. 1992; Käpylä et al. 2006; Pipin and Kosovichev 2013). The obvious drawback of mean-field models is that the individual effects can be adjusted which leads to a great temptation to fine-tune the models. The main advantage of 3D simulations is that this freedom is greatly reduced (although by no means completely eliminated). A major part of the debate regarding the solar dynamo revolves around the relevance of the tachocline.

Unfortunately the advent of 3D simulations with and without tachoclines has not provided conclusive evidence one way or the other. For example, Guerrero et al. (2016) studied both cases with Eulag simulations and found that dynamos operating solely in the convection zone had shorter cycles and intermittent turns-off of activity. On the other hand, dynamos operating at tachocline levels result in long, coherent, magnetic cycles. Whereas the dynamos operating in the convection zone are understood as distributed $\alpha\Omega$ dynamos, those operating at the tachocline may be of $\alpha^{2}\Omega$ type, with the $\alpha$ effect generated by instabilities that extract energy from the magnetic field (e.g., Tayler or buoyancy instabilities; see Guerrero et al. 2019). Furthermore, simulations with (Käpylä 2022) and without (Käpylä et al. 2012; Warnecke 2018) tachoclines from other models produce cyclic dynamos that share some characteristics of the solar cycle. A general conclusion is that long cycles appear to be generated at depth while shorter ones have their origin near the surface (e.g. Käpylä et al. 2016).

Apart from the non-linear dynamo invoked by Strugarek et al. (2017) and Brun et al. (2022), another physical mechanism proposed to be responsible for the equatorward migration include helicity inversion in the deep parts of the convection zone. This is typically encountered in overshoot regions below the convection zone (e.g. Ossendrijver et al. 2001). However, a much deeper helicity inversion was obtained in simulations by Duarte et al. (2016), and which resulted in a change of the propagation direction of the dynamo wave in accordance with the Parker-Yoshimura rule. In these simulations convection was, however, inefficient in the bulk of the convection zone which is not the situation in the solar convection zone. Such reversed helicity configuration can perhaps arise if much of the convection zone is stably stratified as in the proposed entropy rain scenario, but there is currently no simulation that has produced this.

A yet further possibility is that the solar dynamo is driven predominantly by the kinetic helicity as in classical $\alpha^{2}$ dynamos that can lead to equatorward migration if the $\alpha$ effect has a sign change at the equator (Baryshnikova and Shukurov 1987; Rädler and Bräuer 1987). Such helicity profile is expected due to symmetry arguments theoretically, and is a standard outcome in convection simulations (e.g. Brun et al. 2004). The equatorward migrating dynamo wave has been demonstrated by three-dimensional forced turbulence simulations (e.g. Mitra et al. 2010; Warnecke et al. 2011), but no definitive evidence from convection exists.

There have also been attempts to capture the long term modulations in dynamo cycles from simulations. These studies have extended the simulations to cover several tens of cycles corresponding to up to a millenium in solar time (e.g. Passos and Charbonneau 2014; Augustson et al. 2015; Käpylä et al. 2016). These simulations revealed modulation of activity and occasional periods of low activity reminiscent of grand minima (Augustson et al. 2015; Käpylä et al. 2016). Such grand minima states can arise due to an interplay of symmetric and anti-symmetric dynamo modes (e.g. Tobias 1997) or stochastic fluctuations in the buoyancy driving or in the conventional $\alpha$ effect (e.g. Ossendrijver 2000; Brandenburg and Spiegel 2008). However, the modulations and minima in simulations are clearly weaker compared to the solar observations. This is perhaps not too surprising because to run these simulations sufficiently long they need to be done at modest resolutions and cannot therefore be highly supercritical.

### The Sun at Different Ages

During its 4.5 billion years of evolution, the Sun has experienced various changes in its internal constitution, and therefore, the extent of its convection zones and resulting large-scale flows and magnetic fields. In this section we describe numerical simulations of the Sun, or Sun-like stars, corresponding to these evolutionary stages from the formation to the current age.

#### The Pre-Main Sequence Phase

Significant structural changes occurred early in the solar evolution during the pre-main sequence (PMS) stage, as the newly formed object is still contracting. Objects at this stage, with masses similar to the solar mass, are called TTauri stars. While the temperature at the center of the protostar is still increasing, the opacity of the gas is high and the transport of energy occurs entirely due to convection. The actual rotation rate of the Sun in the TTauri phase is unknown, but models can be constructed to characterise it (e.g. Ahuir et al. 2020). Moreover, observations of open clusters have found distributions of rotational periods between roughly 1 and 10 days for solar-like stars with ages around $3\text{ Myrs}$ (see e.g., Gallet and Bouvier 2013).

The large-scale magnetic fields of TTauri stars are predominantly dipolar with field strengths of the order of kG (e.g. Johns-Krull 2007). Fields with a similar topology are also often observed in low mass, fully convective and rapidly rotating M dwarfs (e.g. Kochukhov 2021). Therefore, despite the difference in mass, simulations of TTauri stars and low-mass M dwarfs are, to some extent, comparable. However, the latter will be discussed in detail in Sect. 5.3.3. Following the evolution further, the protoplanetary disc disappears after about $10^{6}-10^{7}$ years since the beginning of the collapse. The protostar continues to contract, and therefore its angular velocity increases. Simultaneously, the star starts to develop a radiative core. Both, the angular velocity and the radiative zone increase before the star reaches the zero age main sequence (ZAMS) after about $5\times10^{7}$ years. As mentioned above, during the TTauri phase the magnetic field of a solar-like star is mainly dipolar. Observations suggest increasing complexity of the magnetic topology once the star develops a radiative zone (Gregory et al. 2012).

There are currently only a few simulation studies that specifically target dynamos in the TTauri and PMS phases of stellar evolution. One such example is the study of Zaire et al. (2017) who considered models in the fully and partially convective phases of PMS evolution. While the differential rotation was more pronounced in the latter evolutionary phase, the resulting quasi-steady predominantly quadrupolar magnetic field configurations were quite similar. A more complete study was performed by Emeriau-Viard and Brun (2017), where five epochs between the TTauri stage and the ZAMS were studied for a $1~M_{\odot}$ star. Each epoch is characterised by a diffrent internal structure and rotation period. The sequence of simulations shows a decreasing dipole contribution to the magnetic field as a function of age. However, even in the early fully convective phase, the dipole constitutes only about ten per cent of the total magnetic energy. In this case the azimuthally averaged large-scale magnetic field is cyclic with poleward migration, reminiscent of the simulations of fully convective M dwarfs (e.g. Brown et al. 2020; Käpylä 2021). The transition between dipole-dominated and multipolar dynamos is discussed in more detail from the perspective of simulations in Sect. 5.3.3.

#### Main Sequence Sun-Like Stars: Rotational Evolution of Differential Rotation and Dynamos

On the main sequence, the rotation rate of stars decreases following the observational Skumanich (1972) law, associated to the loss of angular momentum due to magnetized stellar winds. There have been some speculative ideas about the origin of the non-saturated and saturated regimes of the rotation-activity relationship of stars (e.g. Kawaler 1988; Matt et al. 2015). For instance, Wright et al. (2011) suggested that a turbulent (interface) dynamo is at work in rapidly (slowly) rotating stars. However, the fact that fully convective stars also follow the power law for slow rotation (Wright and Drake 2016) suggests the possibility of a general dynamo theory for all main sequence stars. Nevertheless, to the date, there is no agreement about this theory. One of the main hindrances is the difficulty in observing differential rotation as a function of ${\mathrm{Ro}}$ (e.g. Reinhold and Arlt 2015; Benomar et al. 2018), but a new approach has been recently proposed by Noraz et al. (2022) using Kepler data. Equally difficult is obtaining unambiguous measurements of dynamo cycle periods and systematics as a function of rotation as already discussed. Numerical simulations, on the other hand, can be performed at arbitrary rotation rates corresponding to different ages of the star. Below, we summarize the relevant findings for the differential rotation and dynamos for a solar mass star from its youth to the present age and beyond (see also Noraz et al. 2023).

**Fig. 8:**
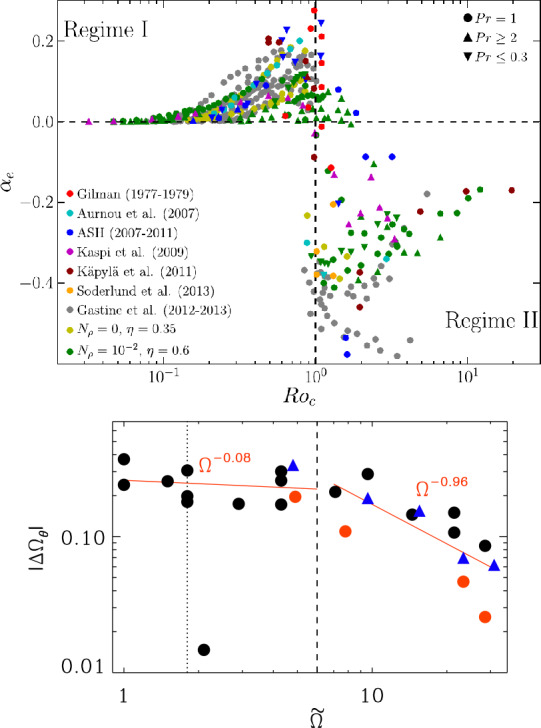
Top panel: Measure of the radial differential rotation $\alpha_{e}=\Delta\Omega/\Omega_{0}$ as a function of ${\mathrm {Ro}}_{\mathrm {c}}$ at the equator for several studies in the literature. Adapted from Gastine et al. (2014). Bottom panel: Modulus of the absolute latitudinal differential rotation from simulations of a solar-like star Viviani et al. (2018), where $\tilde{\Omega}$ is the rotation rate normalized by the solar rotation (©ESO. Reproduced with permission). The dotted line corresponds to the transition of anti-solar to solar-like differential rotation at ${\mathrm {Ro}}_{\mathrm {f}}\approx0.35$ and the dashed line separates the two regimes of differential rotation dependence at ${\mathrm {Ro}}_{\mathrm {f}}\approx0.08$

The Rossby number dependence of large-scale mean flows has been studied in various papers (e.g. Ballot et al. 2007; Käpylä et al. 2011a,b; Guerrero et al. 2013; Gastine et al. 2014; Featherstone and Miesch 2015; Brun et al. 2017). These studies considered different rotation rates for roughly the same structural model resembling the solar interior. Irrespective of the numerical scheme, the results confirmed that the relative radial differential rotation $\Delta\Omega/\Omega$ changes from positive (solar-like differential rotation), for small ${\mathrm {Ro}}_{\mathrm {f}}$, to negative (anti-solar) for large ${\mathrm {Ro}}_{\mathrm {f}}$ (see top panel of Fig. 8, adapted from Gastine et al. 2014), with the transition happening near ${\mathrm {Ro}}_{\mathrm {f}}=1$. In Viviani et al. (2018), the modulus of the absolute differential rotation was also found to decrease rapidly with the rotation rate for ${\mathrm {Ro}}_{\mathrm {f}}\lesssim0.1$; see bottom panel of Fig. 8 and Fig. 8 of Brun et al. (2022). This decrease, however, can be due to low supercriticality of convection at such low ${\mathrm {Ro}}_{\mathrm {f}}$. General consensus from simulations is that for sufficiently rapid rotation the differential rotation is negligibly small. This has implications on the theoretical interpretation of dynamos in the classical mean-field dynamo framework; see Sect. 6. Another characteristic is the appearance of non-axisymmetric convective modes, or active nests, in the rapidly rotating regime, ${\mathrm {Ro}}_{\mathrm {f}}\ll1$ (Brown et al. 2008). Such non-axisymmetric convection has recently been suggested to be the origin of stellar active longitudes (Bice and Toomre 2022). Regarding the structure of convection, it is evident from all simulations that rotation breaks the broad convective cells observed in non-rotating or slowly rotating simulations. Quantitatively, Featherstone and Hindman (2016) found that the harmonic degree where the spectrum has a maximum, $\ell_{\mathrm{peak}}$, scales with the Rossby number as $\ell_{\mathrm{peak}}\sim {\mathrm {Ro}}_{\mathrm {f}}^{-1/2}$ (see also Viviani et al. 2018). This means that the faster the rotation, the smaller the scales where most of the kinetic energy is contained. This applies to regions of the convection zone where ${\mathrm {Ro}}_{\mathrm {f}}\lesssim1$; in the near-surface layers ${\mathrm {Ro}}_{\mathrm {f}}\gg 1$ even in the most rapidly rotating stars, and the size of photospheric convection cells is likely independent of stellar rotation.

As discussed in Sect. 4, the rotation of stars slows down due to magnetic braking as they age. The young Sun was therefore a much faster rotator than what it is today. Simulations of rapidly rotating (${\mathrm {Ro}}_{\mathrm {f}}\lesssim0.1$) young solar-like stars have been performed by several groups using various numerical methods. Quite surprisingly, the results in this parameter regime are rather inhomogeneous: there are three distinct dynamo modes that have been reported from such studies. First, there is the large-scale dipole-dominated solutions (e.g. Gastine et al. 2012; Yadav et al. 2015b; Zaire et al. 2022) that are reminiscent of the geodynamo and those of Saturn and Jupiter. Another outcome is that the large-scale magnetic fields are dominated by a non-axisymmetric $m=1$ mode that often propagates either in retro- or prograde fashion (e.g. Cole et al. 2014; Yadav et al. 2015b; Viviani et al. 2018; Viviani and Käpylä 2021; Navarrete et al. 2022). Finally, some simulations produce predominantly axisymmetric but multipolar large-scale fields in such rapidly rotating setting (e.g. Strugarek et al. 2018). It is unclear why there is such a variety in magnetic field topologies in this regime. A possible cause is that the dynamo is sensitive to relatively minor differences in the boundary conditions (see, e.g. Warnecke et al. 2016) and/or other details of the simulation setups (Orvedahl et al. 2021).

**Fig. 9:**
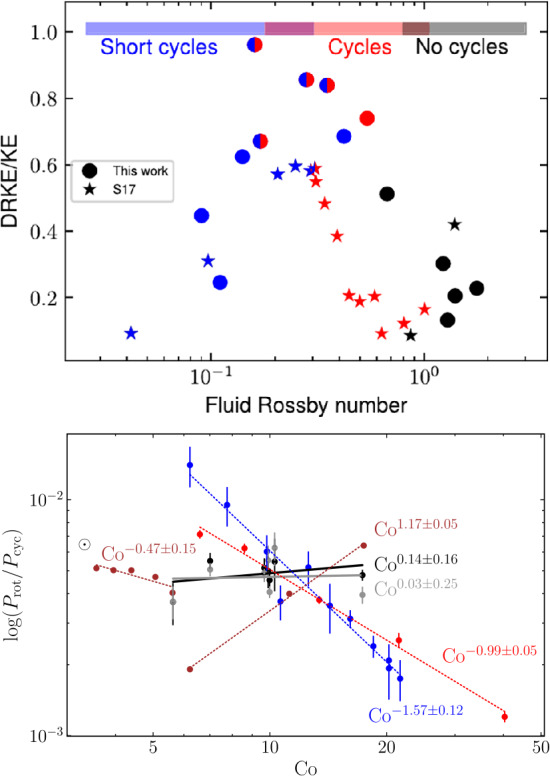
Top panel: Summary of the fraction of differential rotation energy from total kinetic energy as a function of ${\mathrm {Ro}}_{\omega}$ from the simulations of Strugarek et al. (2017) and Brun et al. (2022). The colours of the symbols indicate the type, or the lack of, magnetic cycles. Adapted from Brun et al. (2022). Bottom panel: Comparison of the ratio of the rotation period and the cycle periods as a function of the Coriolis number (${\mathrm {Co}}= {\mathrm {Ro}}_{\mathrm {f}}^{-1}$) from the studies of Strugarek et al. (2018) (blue), Warnecke (2018) (red), Guerrero et al. (2019), and Käpylä (2022). Estimated location of the Sun is indicated by the symbol ⊙. Adapted from Käpylä (2022)

Two further regimes of dynamos can be distinguished on either side of transition between solar-like to anti-solar differential rotation. When rotation is rapid enough such that a solar-like differential rotation is produced, the large-scale fields are predominantly axisymmetric and often cyclic (e.g. Brown et al. 2010; Ghizaru et al. 2010; Käpylä et al. 2012; Nelson et al. 2013; Augustson et al. 2015; Warnecke 2018; Matilsky and Toomre 2020). As mentioned above, in some cases this behavior continues to much more rapid rotation whereas in others non-axisymmetric or dipolar dynamos modes take over. When rotation is slow enough and the differential rotation is anti-solar, the magnetic fields are predominantly axisymmetric and quasi-steady (e.g. Käpylä et al. 2017; Strugarek et al. 2018; Warnecke 2018). In the transition between the two regimes, the dynamo may excite the two modes simultaneously (Viviani et al. 2019). The appearance of cycles appears to be related to the strength of the differential rotation such that long decadal cycles, such as in the Sun, appear in a relatively narrow range of Rossby numbers where the differential rotation is solar-like and it is sufficiently strong (Guerrero et al. 2019; Warnecke and Käpylä 2020; Brun et al. 2022, see the top panel of Fig. 9).

Regardless of the commonalities between different modeling approaches, the simulated magnetic cycles seem to be sensitive to the subtleties of the models, and there is no clear agreement regarding the scaling of cycle periods as a function of rotation as can be seen in the bottom panel of Fig. 9 compiling the results of Strugarek et al. (2018), Warnecke (2018), Guerrero et al. (2019), and Käpylä (2022). Furthermore, none of the scaling laws from simulations seem to unambiguously agree with the observed cycles that are sometime grouped in activity branches with $P_{\mathrm{rot}}/P_{\mathrm{cyc}}\propto{\mathrm{Co}}^{\alpha}$, where $\alpha> 0$ (e.g. Saar and Brandenburg 1999; Brandenburg et al. 2017). It is worth mentioning, however, that the observational results may also present problems and, depending on the used analysis techniques, the distinct activity branches may not even exist (e.g. Bonanno and Corsaro 2022).

As shown in Fig. 8 (top), the amplitude of the differential rotation has maxima on either side of the solar-like to anti-solar transition. The strong shear in the anti-solar regime has been speculated to lead to enhanced magnetic activity (Brandenburg and Giampapa 2018). While this has not been systematically studied, some evidence of enhanced magnetic energy for anti-solar differential rotation has been found in simulations (e.g. Karak et al. 2015; Warnecke and Käpylä 2020; Brun et al. 2022). A much more unambiguous observational fact is that the stellar magnetic activity and magnetic fields saturate for ${\mathrm{Ro}}\lesssim0.1$ (e.g. Reiners et al. 2022, see also Sect. 4). This does not appear to happen in simulations, where the magnetic energy typically increases with rotation even for the fastest rotation considered so far (e.g. Warnecke and Käpylä 2020). Furthermore, the ratio of magnetic to kinetic energy increases roughly proportional to ${\mathrm {Ro}}_{\mathrm {f}}^{-1}$ (Augustson et al. 2019; Warnecke and Käpylä 2020) in accordance with Magneto-Archimedean-Coriolis (MAC) balance. Brun et al. (2022) showed that the large-scale surface fields follow even a steeper increasing trend $\propto {\mathrm {Ro}}_{\mathrm {f}}^{-1.4}$ with decreasing Rossby number. However, this is consistent with stellar observations in the magnetically non-saturated regime (See et al. 2019; Brun et al. 2022). Nevertheless, it is unclear why the simulations deviate from observations in that they do not show indications of saturation when the Rossby number is decreased. However, the magnetic fields of rapidly rotating stars appear to follow a similar scaling law as the geo- and planetary dynamos as well as rapidly rotating convective dynamo simulations (Christensen et al. 2009; Yadav et al. 2013).

Another possibility is that small-scale dynamo action could also play a role in this by diverting energy to smaller scale fields that are more rapidly diffused. It is likely that most rapidly rotating stellar dynamo simulations currently do not have SSDs because of too low ${\mathrm {Re}}_{\mathrm {M}}$ and reduced integral scale of turbulence due to the rotational constraint. This is also manifested by the relative dearth of studies concentrating on the interactions between SSD and LSD. The studies of Käpylä et al. (2017) and Ortiz-Rodríguez et al. (2023) considered simulations of solar-like and fully convective stars, respectively, and where ${\mathrm {Pr}}_{\mathrm {M}}$ was varied while all of the other parameters were fixed. Both studies found that when ${\mathrm {Pr}}_{\mathrm {M}}$ (and consequently ${\mathrm {Re}}_{\mathrm {M}}$) is increased sufficiently such that an SSD is excited, the relatively regular cycles of large-scale fields at low and moderate ${\mathrm {Re}}_{\mathrm {M}}$ are disrupted and differential rotation is severely quenched. On the other hand, Hotta et al. (2016) found that also large-scale fields were amplified when an SSD was excited in comparison to a run where it was absent. The SSD and its interaction with LSD is discussed in more detail in Rempel et al. (2023).

It is also plausible that missing surface physics, such as the lack of spot formation in the simulations contributes to the issue of saturation of magnetism at small ${\mathrm {Ro}}$. There are a handful of global simulations that achieve dynamo action and buoyantly rising flux bundles (Nelson et al. 2013, 2014; Fan and Fang 2014). These buoyant structures are much larger than active regions in the Sun or thin flux tubes that are envisaged to be essential for flux transport dynamos. Furthermore, none of the current simulations produce surface features that are comparable to sunspots. The main reason for this is that the spatial resolution of current global simulations is likely much too coarse to capture the spot formation process self-consistently, irrespective if the origin of spots is buoyant rise of thin flux tubes or some local MHD instability. Instead of trying to solve the entire problem in a single model, Chen et al. (2017) presented a hybrid solution where the suitably scaled magnetic field from a global dynamo simulation from Fan and Fang (2014) was used as a lower boundary condition in a high-resolution Cartesian surface convection simulation that has the resolution to capture spot formation. Coupled modelling efforts such as this are likely to be the best bet in the near future to capture self-consistent dynamo action and spot formation.

### Stars Other than the Sun

Most stars are not like the Sun. A large majority (in our galaxy, anyway) are M dwarfs, which are less massive than the Sun, considerably less luminous (ranging down to about $10^{-3}~L_{\odot}$), and in some cases convective throughout their interiors. At the other end of the H-R diagram, stars more massive than the Sun have convective cores and predominantly stable (radiative) envelopes, accompanied in some cases by thin near-surface convection zones. These high-mass stars can be thousands of times more luminous than the Sun; thus across the main sequence, luminosities vary by a factor of more than a million. These enormous variations in luminosity, and in the geometry and stratification as well, must influence the convection and dynamo action occurring in these stars. In this section, we therefore briefly describe our current understanding of dynamo action in main-sequence stars at lower and higher masses. Our focus here is on what has been revealed by basic theory and by simulations.

From the standpoint of dynamo theory, stars vary not just in their luminosity but also their rotation rate, their geometry, their stratification, and their microphysics. For example the core of a massive star is only weakly stratified, whereas an M dwarf (or the convective envelope of a Sun-like stars) is much more strongly stratified in density and temperature. The microscopic diffusivities vary in such a way that ${\mathrm{Pr}}_{\mathrm{M}}$ is very small in many stellar convection zones but greater than unity in some (e.g. Augustson et al. 2019; Jermyn et al. 2022). Many of these variations are intertwined. For example, the influence rotation has on the convection is typically encapsulated by some version of the Rossby number ${\mathrm{Ro}}\sim P_{\mathrm{rot}}/\tau_{\mathrm{conv}}$; because $\tau_{\mathrm{conv}}$ depends on the flow velocity, the rotational influence can vary from star to star (and with radius within a given star) even if the rotation period is constant.

Faced with the bewildering array of possible variations in all of these parameters, would-be simulators tend to try one of two different approaches. One is to focus on a particular physical effect – stratification, for example – and try to elucidate how this affects convection, differential rotation, and magnetism, typically in an idealized setting (e.g., a Cartesian layer). Another is to attempt to model a given astrophysical object in some detail, adopting spherical coordinate systems and stratifications (of density, temperature, or entropy) that mimic those in a 1D stellar model. In neither approach is it possible for simulations to approach the actual parameter regimes attained in real stars, although some balances can be recovered that help in understanding, e.g. the type of differential rotation that occur in real stars. Before diving into a “by object” discussion, we turn first to a high-level overview of the effects of rotation, stratification, and geometry on dynamo action in general. Then we turn to summaries of how these effects play out in specific types of stars.

#### Effects of Rotation, Stratification, and Geometry in Stellar Models

All stars rotate, all have some level of density stratification, and all convect in a spherical geometry. Here we provide a very brief overview of how each of these effects likely influence the flows and magnetism. Perhaps the clearest message from the last few decades of research on convection and dynamo action is, “rotation matters a lot.” It affects the convective flows, it affects their transport of heat and angular momentum, and it affects the magnetism these flows can build. Some of these effects (e.g., how rotation affects magnetic field morphology) are understood only qualitatively, whereas for others (e.g., how it affects heat transport) there is now a more quantitative picture.

To begin, consider the influence of rotation on the convective flows in the absence of magnetism. It is well-known that rotation tends to stabilise a system against convection, increasing the critical Rayleigh number for onset (${\mathrm{Ra}}_{\mathrm{c}} \propto{\mathrm{Ek}}^{-4/3}$, with ${\mathrm{Ek}}$ the Ekman number defined previously), while the most unstable wavenumber shifts to higher wavenumber ($k$) with more rapid rotation rate (lower ${\mathrm{Ek}}$) (e.g. Chandrasekhar 1961). (Less obviously, consider a numerical simulation not too far from onset – more specifically, one for which “diffusion free” scalings do not yet apply – with, for example, a fixed heat flux or temperature contrast, and also some fixed rotation rate. In this system changing the numerical diffusivities – or in an implicit LES simulation changing the resolution – will also affect the rotational influence, via changing ${\mathrm{Ek}}$, and so also the flows.) In a global spherical geometry, whenever rotation play a major role in the dynamics the most prominent convective modes close to onset are wave-like convective rolls, aligned with the rotation axis and arising from the conservation of potential vorticity, variously called “thermal Rossby waves,” “Busse columns,” or “banana cells” depending on the community (see, e.g. Busse 1970; Gilman 1977; Busse 1983; Featherstone and Hindman 2016; Bekki et al. 2022). The systematic prograde tilt of these cells, and the associated Reynolds stress, plays a major role in redistributing angular momentum in most global-scale simulations of stellar convection; the differential rotation is then also a strong function of rotation rate (Rossby number), as summarized elsewhere (e.g. Gastine et al. 2014).

The heat transport by the convection is also strongly influenced by rotation: for a given fixed heat flux, the convection tends to become “less efficient” as the rotation rate increases – that is, it requires a larger entropy gradient to carry the same flux (see, e.g., discussions in Stevenson 1979; Aurnou et al. 2020). Quantitatively, several theoretical approaches supported by numerical simulations – including appeals to a balance between Coriolis, inertial, and buoyancy forces (so-called “CIA balance”) (e.g Vasil et al. 2021), “rotating mixing length” theory (Stevenson 1979; Barker et al. 2014; Currie et al. 2020) asymptotic theory at low Rossby number (Julien et al. 2012) – yield the same diffusion-free dependence of key quantities, like the temperature gradient, when rotation is rapid enough. In these models the amplitude of the convective motions goes down at higher rotation rates, the temperature gradient goes up, and the typical wavenumber of the flow increases, with important consequences for the dynamo.

The dependence of dynamo action itself on rotation is less quantitatively understood, though it is still possible to make some broad statements that are supported by theory and simulation. When rotation is dynamically significant, it is widely thought to affect the strength and morphology of dynamo-generated magnetic fields as discussed in more detail in Sect. 5.2.2. (see also, e.g., Schrinner 2013; Augustson et al. 2019; Orvedahl et al. 2021). Furthermore, because the envelope convection zones of low-mass stars are strongly stratified (in density and temperature), many authors have also sought to understand what role this stratification plays in determining various properties of the flows and magnetism. We mention here only a few. Broadly, the presence of density stratification breaks the up-down symmetry in Boussinesq systems, so it changes the convective dynamics – and also presumably the magnetism – in a variety of ways. Strongly stratified systems tend to have strong, narrow downflows and broader, weaker upflows (e.g. Hurlburt et al. 1984; Cattaneo et al. 1991); their energy dissipation budget is very different (e.g., Hewitt et al. 1975; Currie and Browning 2017); they can establish different profiles of kinetic helicity (Duarte et al. 2016) and may drive different types of zonal flows (e.g., Glatzmaier et al. 2009). If diffusion-free scalings (like mixing-length theory) apply, or simply on dimensional grounds, the velocity of the convective flows is expected to vary in amplitude across a stratified convection zone, in a way not found in Boussinesq systems. This basic fact has profound consequences for the convective dynamics in a star like the Sun, since we then expect regions near the photosphere (which are very low-density) to undergo such rapid convective motions that their Rossby number is enormous (i.e., the influence of rotation is negligible there); meanwhile the deeper flows must be influenced by rotation, at least within some finite distance of the transition to the radiative interior. The presence of density stratification may make it harder to sustain a strong global-scale dipole in some regimes (e.g., Gastine et al. 2012), but it is also clear that highly ordered fields are still realizable even when the stratification is strong (see discussions in Raynaud et al. 2015, Menu et al. 2020, Zaire et al. 2022).

Relatively little work has directly addressed the influence of the *geometry* of the convection zone, while keeping other factors constant. We briefly note only a few specific results. Goudard and Dormy (2008) showed that, in rotating Boussinesq MHD simulations with fixed temperature boundaries, changing the aspect ratio of the domain (i.e., changing the depth of the convection zone) had a strong effect on the nature of their dynamo solutions. In deep domains, they found steady “Earth-like” dipolar solutions; if the convection zone was gradually made thinner, they found a transition to “Sun-like” dynamo wave solutions. Separately, Camisassa and Featherstone (2022) have recently investigated the role of geometry in determining the Reynolds stresses – and hence the differential rotation – in anelastic simulations of rotating convective envelopes. They argue that the well-known transition from solar to anti-solar differential rotation occurred when columnar convective structures attained a diameter roughly equivalent to the shell depth. In the sections that follow, we explore how these different dynamical trends play out in models of specific stars.

#### Dynamo Action in High-Mass Stars: Convective Cores and Radiative Envelopes

Massive stars on the main sequence possess convective cores, with a predominantly stable radiative envelope above this. The high luminosities established by nuclear fusion in these stars mean that we expect the convection to be vigorous; the accompanying high temperatures mean that we expect these convection motions should generally act as magnetic dynamos, since all plausible estimates of the magnetic Reynolds number ${\mathrm{Re}}_{\mathrm{M}}$ are very high; see Table 1. It may well be that dynamo action is occurring in the radiative layers as well, as discussed below. Both the flows and fields have lately been targets of intense scrutiny – partly because asteroseismology has begun to provide powerful new constraints on this topic, particularly for evolved stars (see, e.g., Stello et al. 2016, Ji et al. 2023) and also because of the implications these hold for later stages of stellar evolution (e.g., Fuller and Ma 2019). In this section, we briefly review what has been learned by simulations focusing on these types of stars. A more thorough description can be found in Brun and Browning (2017).

Early global simulations covering some aspects of the problem include Kuhlen et al. (2003), Browning et al. (2004), and Brun et al. (2005). Taken together, these papers provided the first numerical estimates of the flows and magnetism that might be generated in 3D massive star cores. They also gave some estimates of the overshooting and penetration from the convective zone into the surrounding stable envelope, the gravity wave response there, and the differential rotation arising from the interplay of convection, magnetism, and rotation. Later work has pushed towards more realistic (turbulent) flows, has examined a variety of initial states (e.g., strong magnetic “fossil” fields) and srutinized each facet of this complicated problem more systematically. We refer the interested reader to Meakin and Arnett (2007), Featherstone et al. (2009), Gilet et al. (2013), Rogers (2015), Augustson et al. (2016), Edelmann et al. (2019), Breton et al. (2022), and Baraffe et al. (2023) as a representative sample of relevant work.

There has lately also been sustained interest in the topic of magnetism generated by dynamo action in the radiative envelope, typically as a result of the interaction between shear and magnetic instabilities (e.g., Spruit 1999). Numerical work – beginning with Braithwaite (2006) and followed by many others since (e.g., Zahn et al. 2007, Duez et al. 2010, Jouve et al. 2015, Vidal et al. 2018, Petitdemange et al. 2023, Ji et al. 2023) – has examined the circumstances under which such dynamo action could occur, and the strength of the resulting fields.

Taken as a whole, these simulations are unequivocal about a few points. Very strong fields can plausibly be established by dynamo action in the cores of some massive stars, whether rotation is dynamically significant or not; for example, because the convection is rapid, even the “equipartition-scale” field (equating $\rho u_{\mathrm{conv}}^{2}$ with $B^{2}/(4\pi)$ and assuming MLT scalings for the convective velocity $u_{\mathrm{conv}}$) would suggest $B \ge10^{6}$ G in the interior of a B-type star. Some of these stars rotate very rapidly, and the saturation strength of the field in this case is less clear (see, e.g., discussions in Augustson et al. 2019), but it seems likely that strong fields are the norm rather than the exception. The differential rotation established within the core is less certain, because it is surely influenced by the strength of the magnetism – which is likewise influenced by the shear – but broadly these simulations appear to obey trends similar to those in simulations of convective envelopes. “Solar-like” differential rotation (i.e., with a prograde equator) is established when rotation is dynamically significant and the magnetism is not too strong; “anti-solar” profiles arise when the influence of rotation is weaker; magnetism reduces the shear and may yield solid-body rotation if it grows strong enough. Convective motions overshoot into the radiative envelope, though the extent of this effect is still being actively investigated (e.g., Anders et al. 2022b, Baraffe et al. 2023), and excite a substantial gravity-wave response that may be detectable even in main-sequence stars (e.g., Breton et al. 2022).

Within the radiative zone itself, the latest simulations (Petitdemange et al. 2023, Ji et al. 2023) now appear to be capturing some aspects of the long-envisioned “Tayler-Spruit” dynamo. This differs in some important respects from the picture originally envisioned by Spruit and debated in Zahn et al. (2007); for example, in the Petitdemange et al. (2023) simulations only *subcritical* dynamo action is found, alongside some other dynamo instability. The saturation amplitude of the field is still very much under debate; see discussions in Spruit (1999), Fuller et al. (2019), and Ji et al. (2023).

#### Low-Mass Stars and the Transition to Full Convection

Main-sequence stars less massive than the Sun have deeper convective envelopes (as a fraction of the total stellar radius). Below a mass of about $0.35~M_{ \odot}$ stars are – in standard 1D models – convective throughout their interiors. This transition occurs at a spectral type of around M3, so “M dwarfs” in general hold special interest theoretically, as probes of the various roles that rotation and stratification play in the dynamo. They are also interesting astronomically: the large majority of stars in our galaxy are M dwarfs, and they are popular targets in the quest to find and characterise exoplanets (e.g., Trifonov et al. 2018).

Here, we briefly review attempts to model these stars numerically. In terms of fundamental fluid dynamics, these stars have many similarities with pre-main sequence stars (which are also fully convective, but can have different internal heating profiles and rotational constraints), so in places our discussion parallels that in Sect. 5.2.1. In some other respects the flows in these objects resemble those in giant gaseous planets, so we also draw comparisons to the extensive literature on planetary dynamos. Finally, there are also many similarities with the flow in massive-star cores, which share the same geometry (i.e., a full sphere of convection) but are much less strongly stratified than a main-sequence M dwarf; see Table 1.

The first 3D MHD simulations that aimed specifically to model low-mass fully convective stars were reported in Dobler et al. (2006). They used the Pencil Code, solving the fully compressible equations to model a spherical star (established via volumetric heating and cooling terms) embedded in a Cartesian grid. These first models included only a fairly weak density stratification (with $\rho$ at the center of the star about a factor of three greater than at its photosphere). Later, Browning (2008) conducted the first anelastic simulations (with the ASH code) that mimicked low-mass M dwarfs, including a stronger density stratification (about a factor of 100 across the deep spherical shell) and more complex flows. Subsequent work has sampled much lower diffusivities, more extreme density stratifications, and varying rotational influences. We note in particular the simulations of Gastine et al. (2012) and Yadav et al. (2015, 2016), modelling anelastic dynamos in a deep spherical shell with the MagIC code; Brown et al. (2020), who considered a full spherical geometry (i.e., in spherical coordinates but with no singularity at $r=0$) using the Dedalus framework; Käpylä (2021), who considered “star-in-a-box” models akin to those of Dobler et al. (2006) but in a substantially different parameter regime; and Bice and Toomre (2020, 2022), modelling deep (anelastic) shells of convection with the Rayleigh code.

Although there are many differences between these models, some broad trends are now reasonably clear, and largely parallel those realised in other objects and geometries (as discussed elsewhere in this review). When magnetism is weak or absent, both “solar” and “anti-solar” differential rotation can be realised, depending upon the rotational influence (i.e., some version of the Rossby number). When magnetism is strong, it reduces this differential rotation and – if the field gets strong enough – can essentially eliminate it, leading to solid-body rotation. The spatial structure of the field – e.g., the fraction of the magnetism in axisymmetric components, or the strength of the dipole or quadrupole moment – is intertwined with rotation, shear, and density stratification in a complex manner.

When rotation is strong and shear is weak, the field tends to develop a large-scale dipolar component; see discussions in, e.g., Gastine et al. (2012), Schrinner et al. (2012), Yadav et al. (2015), and an array of related works modeling planetary dynamos with weak stratifications (e.g., Christensen and Aubert 2006, Schwaiger et al. 2021). In real stars, presumably any large-scale field generation is accompanied by vigorous SSD, so the overall field likely consists of a very wide range of scales. The dipolar solutions are, at least in some parameter regimes, more difficult to realise when the density stratification is strong and when the convective supercriticality is high (e.g., Gastine et al. 2012); but there are now multiple examples of highly-stratified, vigorous convection that exhibit strong dipoles (e.g., Yadav et al. 2015), including some at surprisingly modest rotational constraints (e.g., up to ${\mathrm{Ro}}\sim0.4$ in Zaire et al. 2022). The question of what exactly delineates these states from one another is a topic of very active investigation (see, e.g., discussions in Menu et al. 2020, Tassin et al. 2021, Zaire et al. 2022).

When shear is also present, a variety of solutions are possible, including propagating dynamo waves. Examples abound; see, for example, Yadav et al. (2016), Käpylä (2021), and Bice and Toomre (2022). Again, it seems reasonably clear that the rotational influence is the most crucial control parameter, but many details remain unclear. Other topics of active interest include the prevalence of non-axisymmetric features in the field (e.g., Bice and Toomre 2022, Käpylä 2021), or modes in which the bulk of the magnetism is confined to one hemisphere (e.g., Gallet and Pétrélis 2009, Gastine et al. 2012, Brown et al. 2020). Furthermore, Käpylä (2021) reported that the qualitative succession of dynamo modes as a function of rotation appears to be the same in simulations of fully and partially convective stars: when rotation increases, the predominantly axisymmetric steady and cyclic solutions at slow rotation give way to non-axisymmetric dynamos at rapid rotation. In these models a similar succession happens with the cycles, so that for moderate rotation the dynamo waves typically propagate in latitude; when rotation is more rapid, the large-scale magnetic structure drifts in longitude.

## Connections to Mean-Field Dynamo Theory

Finding out which physical processes lead to the observed magnetic field evolution in 3D convective dynamo simulations is very challenging. An often-used approach is to interpret the outcome of the simulations in terms of mean-field dynamo theory. Mean-field theory provides a well-established theoretical foundation, which can be used to analyse the complex 3D simulation in a simplified way (e.g. Krause and Rädler 1980; Brandenburg and Subramanian 2005); see also Brandenburg et al. (2023). Technically this is done by computing mean-field transport coefficients from 3D simulations and using them in a corresponding mean-field model. This approach has been successfully used to pinpoint the cause of magnetic field evolution in many simulations. In mean-field theory the magnetic and velocity fields are divided into a mean or averaged part and a fluctuation, e.g. $\boldsymbol {B}=\overline {\boldsymbol {B}}+ { \boldsymbol {B}}^{\prime}$. Only the mean fields are explicitly solved for, whereas the (correlations of) fluctuations are parameterized in terms of the mean. Azimuthal averages are often used for solar and stellar dynamos such that the resulting mean field is axisymmetric. However, such an average is not well suited for rapidly rotating stars, where non-axisymmetric $m=1, 2$ modes often dominate (Viviani et al. 2018). Applying the mean-field approach to the induction equation leads to the emergence of an additional term, the electromotive force $\overline {\boldsymbol {\mathcal {E}}}=\overline{{\boldsymbol{u}}^{\prime}\times { \boldsymbol {B}}^{\prime}}$. In mean-field dynamo theory this term is parameterized in terms of the $\overline {\boldsymbol {B}}$ and its gradients, assuming that the mean fields vary slowly in space and time (Krause and Rädler 1980),

28 $$ \overline{\mathcal{E}}_{i}= a_{ij} \overline{B}_{j} + b_{ijk} \frac{\partial\overline{B}_{j}}{\partial x_{k}} + \cdots, $$

where the dots represent higher order derivatives that are most often neglected. The tensors $\boldsymbol {a}$ and $\boldsymbol{b}$ can then be further divided into symmetric and anti-symmetric parts (e.g. Rädler 1980), yielding an equivalent representation

29 $$ \overline {\boldsymbol {\mathcal {E}}}=\boldsymbol {\alpha }\boldsymbol{\cdot} \overline {\boldsymbol {B}}+\boldsymbol {\gamma }\times \overline {\boldsymbol {B}}-\boldsymbol {\beta }\boldsymbol{\cdot}( \boldsymbol {\nabla }\times \overline {\boldsymbol {B}}) -\boldsymbol {\delta }\times(\boldsymbol {\nabla }\times \overline {\boldsymbol {B}}) -\boldsymbol {\kappa }\boldsymbol{\cdot}(\boldsymbol {\nabla }\overline {\boldsymbol {B}})^{(s)}, $$

where $(\boldsymbol {\nabla }\overline {\boldsymbol {B}})^{(s)}$ is the symmetric part of the magnetic field gradient tensor. The coefficients $\boldsymbol {\alpha }$ and $\boldsymbol {\beta }$ are rank two tensors, $\boldsymbol {\gamma }$ and $\boldsymbol {\delta }$ are vectors, and $\boldsymbol {\kappa }$ is a rank three tensor. These coefficients can be associated with different turbulent effects important for the magnetic field evolution: the $\alpha$ effect (Steenbeck et al. 1966) leads to field amplification via helical flows; $\gamma$ describes the turbulent pumping, which acts like mean flow (e.g. Rädler 1968; Roberts and Soward 1975); $\beta$ describes turbulent diffusion; and the $\delta$ effect, also known as the Rädler effect (Rädler 1969) or the shear-current effect (e.g. Rogachevskii and Kleeorin 2003), can lead to dynamo action in non-helical turbulence in the presence of shear; and finally, the $\kappa$ effect, whose physical interpretation is currently unclear.

The main challenge is to determine these coefficients from a 3D global convective dynamo simulation. The ultimate goal is to be able to reproduce the magnetic field solution with a mean-field model using the obtained coefficients.

### Using Proxies Based on Flow and Magnetic Field Properties

The simplest approach to compare 3D convection simulations with mean-field theory is to use approximate proxies of turbulent transport coefficients based on the flow and magnetic field properties. Assuming isotropy and homogeneity and applying the second order correlation approximation (SOCA), the turbulent transport tensors reduce to scalars $\alpha_{\mathrm {K}}$ and $\beta$, (e.g. Krause and Rädler 1980; Brandenburg et al. 2023)

30 $$\begin{aligned} \alpha_{\mathrm{K}} = -{\frac{1}{3}} \tau \overline{{ \boldsymbol {\omega }}^{\prime}\boldsymbol{\cdot} {\boldsymbol{u}}^{\prime}},\ \ \ \beta = {\frac{1}{3}} \tau\overline{\boldsymbol{u}^{\prime2}}, \end{aligned}$$

where $\tau$ is the correlation time of the flow, and where ${ \boldsymbol {\omega }}^{\prime}=\boldsymbol {\nabla }\times {\boldsymbol{u}}^{\prime}$. The expressions of $\alpha_{\mathrm{K}}$ and $\beta$ are valid in the kinematic regime where the back-reaction of the magnetic field on the flow is neglected. In a more general approach, the minimal tau-approximation, this backreaction is retained and this leads to an additional magnetic contribution to the $\alpha$ effect (Pouquet et al. 1976; Blackman and Field 2002)

31 $$\begin{aligned} \alpha_{\mathrm{M}}={\frac{1}{3}} \tau\overline{\rho}^{-1} \overline{{ \boldsymbol {J}}^{\prime}\boldsymbol{\cdot} { \boldsymbol {B}}^{\prime}}, \end{aligned}$$

where ${ \boldsymbol {J}}^{\prime}=\mu_{0}^{-1}\boldsymbol {\nabla }\times { \boldsymbol {B}}^{\prime}$. $\alpha_{\mathrm{M}}$ can be interpreted as a consequence of magnetic helicity conservation (see, e.g. Brandenburg et al. 2023). This approach has been used in many simulation studies to interpret the magnetic field evolution (Charbonneau 2020). For example, Warnecke et al. (2014) used the $\alpha$ proxy to conclude that the equatorward migration found in their, and in previous work, is due to an $\alpha\Omega$ Parker dynamo wave driven by a region of negative radial shear. Similarly, Duarte et al. (2016), explained the equatorward migration in their simulations by the inversion of $\alpha$ and positive radial shear. Guerrero et al. (2016, 2019) concluded that the dynamos in their simulations with tachoclines were driven by $\alpha_{\mathrm{M}}$ below the convection zone. However, it is necessary to bear in mind the approximate nature of analyses based on proxies: often the agreement between the simulation and the behavior suggested by the proxy is qualitative at best and the 3D simulations contain a rich variety of non-linear interactions that are omitted in such analyses.

### Direct Measurements of Coefficients

The alternative is to measure the coefficients in Eq. (29) directly from simulations. There are currently two commonly used methods for this. First, $\overline {\boldsymbol {B}}$ and $\overline {\boldsymbol {\mathcal {E}}}$ from the 3D dynamo simulation can be used to fit for the turbulent transport coefficients in Eq. (29) using, e.g., multidimensional regression method or singular value decomposition (SVD) (Brandenburg and Sokoloff 2002; Racine et al. 2011). On the other hand, the test field (TF) method uses a sufficiently large number of linearly independent *test fields*, that do not back-react on the solution, and evolves the corresponding ${ \boldsymbol {B}}^{\prime}$ and $\overline {\boldsymbol {\mathcal {E}}}$ for each. Then it is possible to unambiguously invert for the coefficients in Eq. (29) (Schrinner et al. 2005, 2007). Both of these methods are, at best, only as good as the approximate equation (29). The validity of the results needs to be tested by inserting the derived coefficients back into Eq. (29) and to mean-field models to determine how faithfully they capture the $\overline {\boldsymbol {\mathcal {E}}}$ and time evolution of the mean field in the 3D simulation.

The SVD method has the issue that Eq. (29) is underdetermined: there are 27 unknown parameters and only three components of $\overline {\boldsymbol {B}}$ and $\overline {\boldsymbol {\mathcal {E}}}$. This is typically overcome by considering the time dependence of $\overline {\boldsymbol {B}}$ and $\overline {\boldsymbol {\mathcal {E}}}$ leading to an overdetermined system. Furthermore, if $\overline {\boldsymbol {B}}$ does not vary in time the SVD method has problems to converge. Despite these difficulties this method has been used to explain the dynamos in several simulations (e.g. Racine et al. 2011; Augustson et al. 2013, 2015). Simard et al. (2016) found that the coefficients related the gradients of $\overline {\boldsymbol {B}}$ ($\boldsymbol {\beta }$, $\boldsymbol {\delta }$, $\boldsymbol {\kappa }$) are much less important than $\boldsymbol {\alpha }$ and $\boldsymbol {\gamma }$. Furthermore, Simard et al. (2013) could reproduce the mean-field evolution of 3D global simulation using $\boldsymbol {\alpha }$ and $\boldsymbol {\gamma }$ determined with the SVD method, basically assuming it is an $\alpha^{2}\Omega$ dynamo. However they had to assume a higher turbulent diffusivity than what was measured. In follow-up studies the authors could explain and reproduce a dual dynamo action (Beaudoin et al. 2016) and generate Grand Minima-like events by including $\alpha$ quenching (Simard and Charbonneau 2020). Recently, Shimada et al. (2022) analysed the simulations of Hotta et al. (2016) with the SVD method and found that the turbulent diffusion decreases with increasing ${\mathrm{Re}}_{\mathrm{M}}$. However, it is unlikely that Eq. (29), and hence the SVD method, is valid at high ${\mathrm{Re}}_{\mathrm{M}}$ where a SSD is excited.

**Fig. 10:**
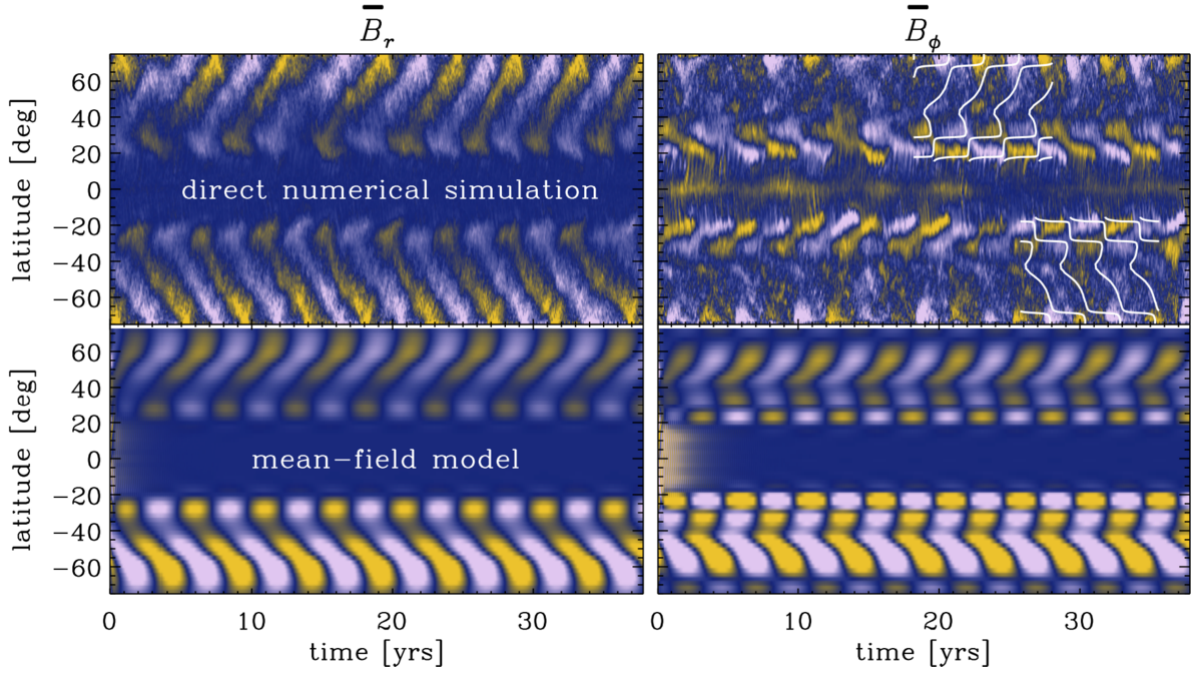
Comparison of direct numerical simulations of Warnecke (2018) and Warnecke and Käpylä (2020) (top) with a corresponding mean-field model (bottom) where the turbulent transport coefficients have been obtain with the test field method. The radial (left) and toroidal (right) mean field is shown as a function of latitude and time. The white contours on the top right panel indicate the corresponding field of the mean-field model. Adapted from Warnecke et al. (2021)

In the TF method, a set of 9 linearly independent test fields are used to uniquely determine the 27 unknowns. First developed for the geodynamo (Schrinner et al. 2005, 2007, 2011, 2012; Schrinner 2011), it has subsequently been used for many solar and stellar dynamo simulations (Gent et al. 2017; Warnecke et al. 2018; Warnecke 2018; Viviani et al. 2019; Warnecke and Käpylä 2020). An important result is that the turbulent pumping is typically larger than the meridional circulation in global convective dynamo simulations, rendering the flux-transport dynamo scenario unlikely in those cases (Warnecke et al. 2018),

because the total (meridional plus turbulent) advection generally does not have closed streamlines. The conclusion of Warnecke et al. (2014) that the equatorward migration in these kind of simulation is explained by a Parker dynamo wave was confirmed with the $\alpha$ effect from the TF method (Warnecke et al. 2018, 2021). This was later used to explain the cycle period dependence on rotation in 3D dynamo simulations (Warnecke 2018). Gent et al. (2017) analyzed the simulations of Käpylä et al. (2016) and found that the turbulent transport coefficients – particularly $\boldsymbol {\gamma }$ – vary significantly during long-term modulation of the cyclic mean magnetic field. In the work of Viviani et al. (2019), the first cyclic dynamo in the anti-solar differential rotation regime was explained to be of $\alpha^{2}\Omega$ type using test-field coefficients. Furthermore, Warnecke and Käpylä (2020) studied the transport coefficients as functions of rotation rate and found that $\alpha\Omega$ dynamos, appear to be possible in a relative narrow range in ${\mathrm{Ro}}$. The trace of $\boldsymbol {\alpha }$ agrees with $\alpha_{\mathrm {K}}$ in pattern and amplitude in a Rossby number range spanning three orders of magnitude (Warnecke and Käpylä 2020). As a result of the test-field analysis (Warnecke and Käpylä 2020) a magnetic influence on $\alpha$ as described in Eq. (31) could be ruled out in their simulations. Putting all the turbulent transport coefficients into a mean-field model, the evolution of the mean magnetic field of the 3D simulation was reproduced in terms of period and pattern (Warnecke et al. 2021); see Fig. 10. Notably the full spectrum of coefficients was needed to fully reproduce the field evolution. This suggests that all of the turbulent mean-field effects play important roles in this simulation which is a good representation of current global dynamo simulations. Furthermore, the authors concluded that the assumptions of Eq. (29) are reasonably well justified in the simulations (Warnecke et al. 2021) given that the $\boldsymbol {\mathcal {E}}$ is also reproduced reasonably satisfactorily (Warnecke et al. 2018; Viviani et al. 2019).

### Remaining Issues

One of the important issues is that the results of the two methods, SVD and TF, do not fully agree with each other. The SVD method seems to produce satisfactory results for EULAG-MHD simulations (Simard et al. 2013; Beaudoin et al. 2016; Simard and Charbonneau 2020). However, Warnecke et al. (2018) showed that the coefficients determined with the TF and SVD methods give quite different results and that the SVD coefficients related to the derivative of $\overline {\boldsymbol {B}}$ are indeed less important. This discrepancy raises questions regarding the overlap of the validity ranges and the underlying assumptions of both methods. A possible reason for the discrepancy is that non-locality (in space and/or time), which is neglected in Eq. (29), plays a role: the SVD uses the actual field whereas in TF only very large-scale gradients of test fields are retained. On the other hand, the actual magnetic fields the SVD uses are not necessarily linearly independent, leading to errors in the inversion.

SVD and TF methods reveal that turbulent transport effects play an important role in the dynamic and evolution of the large-scale magnetic field. Warnecke et al. (2021) showed that it is necessary to include practically all of the possible turbulent effects to reproduce the 3D simulation results in detail. This makes the corresponding mean-field models quite complex which to a certain extent defeats the purpose of mean-field modelling where the hope has been to capture the large-scale behavior of complicated 3D systems by a much simpler lower-dimensional model. However, the complexity of dynamos operating in these simulation also hints in the direction that the Sun and other stars are more complex than simple mean-field models including the Babcock-Leighton model can describe.

Another issue is related to the appearance of the SSD instability when global simulations reach more realistic high-${\mathrm{Re}}_{\mathrm {M}}$ regimes. In this case Eq. (29) is no longer valid, because a contribution to $\overline {\boldsymbol {\mathcal {E}}}$ that is independent of $\overline {\boldsymbol {B}}$ is possible, and non-linearity due to ${ \boldsymbol {B}}^{\prime}$ becomes important. If small-scale magnetic fields due to the SSD are dynamically important, neither the SVD nor TF method will work in their current form. Efforts to generalize the TF method to incorporate the effect of the SSD have been taken by Käpylä et al. (2022), leading to four flavours of the method in that regime. Although these flavours should in principle agree, this is not always the case, especially for high ${\mathrm{Re}}_{\mathrm{M}}$. Hence, it is of great importance to extend the SVD and TF methods to more realistic parameter regimes to incorporate effects such as the SSD.

## Conclusions and Future Prospects

A central challenge in simulating Solar or stellar dynamos is that, as discussed above, the interiors of real stars are characterised by extremely low diffusivities (of momentum, heat, and magnetism), and possess motion and magnetism over an extraordinarily broad range of spatial and temporal scales. No simulation, now or in the near future, can capture all these scales simultaneously. The hope of many modelers, though, is that at least *some* aspects of the dynamics, particularly on the largest scales, may become independent of the small-scale details at high enough resolution (low enough diffusivity). There is considerable debate – even amongst the authors of this review! – about the extent to which present-day simulations are nearing this diffusion-free, resolution-independent regime, and reasons for both optimism and pessimism. Here, we briefly highlight a few of these.

Current global dynamo simulations of stars routinely capture solar-like differential rotation and cyclic magnetism. Sometimes these models also reproduce equatorward migrating activity akin to the Sun. This occurs at a Rossby number regime where differential rotation is relatively strong. These results seem to be fairly robust irrespective of the numerical method or other details of the simulations. Also the theoretical understanding of the physical mechanisms driving the magnetism has developed significantly in the recent years with more advanced analysis tools such as the test-field method, full energy transfer and field production (electromotive force) analysis, and with direct comparisons to mean-field models.

Although our understanding of the physics of convection and resulting dynamo action has increased, new challenges have also been encountered. The most intriguing of these is the fact that current simulations struggle to reproduce solar convection and the resulting differential rotation at the solar luminosity and rotation rate. Given that this is a necessary requirement to get the dynamo right it is not a huge surprise that reproducing the solar dynamo remains challenging. This “convective conundrum” is the modern equivalent to the “dynamo dilemma” of the 1980s. The latter lead to a revival of old, and the conception of new, ideas about solar and stellar dynamos and a similar process is at work with respect to solar and stellar convection at the moment. The various ideas related to solving the conundrum include entropy rain and deep weakly subadiabatic convection, the influence of strong small-scale magnetism, and rotationally constrained deep convection. Research on this topic is very active and evolving rapidly, and, far from being stumped by the challenge posed by the convective conundrum, activity in the modelling of stellar convection and dynamos has instead been invigorated.

A key issue with the Sun is that even though the deep convection zone is highly likely rotationally dominated with ${\mathrm{Ro}}\ll1$, there are many scales in the upper convection zone and near the surface where the rotational influence on the flow is weak. The Sun is also perhaps close to the transition to anti-solar differential rotation, which has some observational support, making it difficult to maintain a solar-like differential rotation profile if the simulations are not sufficiently near the correct parameter regime. Identifying the correct force balance prevailing in the solar convection zone is therefore key to this problem. With this information, simulations can be designed such that they follow a path leading to the correct balances and hopefully to solar-like results. This is a practice adopted in simulations of the geodynamo and perhaps a similar approach can be adopted for the Sun.

On the other hand, the issue regarding rotational influence is not as severe in stars that rotate more rapidly than the Sun and which are further away from the solar-like to anti-solar differential rotation transition. There are indications that simulations capture the characteristics of dynamos, such as non-axisymmetric large-scale fields, and dipole dominated dynamos in M dwarfs, in such stars more accurately. Although this is encouraging, we should also bear in mind that the observational data from other stars is not as accurate and detailed as the data we have from the Sun.

A common characteristic of all of the current simulations is the fact that it is not possible to model the surface layers, where the density drops vertiginously, accurately enough. This could be one of the reasons why none of the current simulations form spots that could play a role in the dynamo process via a Babcock–Leighton type effect, and their magnetic activity does not become independent of rotation at sufficiently low ${\mathrm{Ro}}$. Self-consistent spot formation has not been reported even in local simulations to say nothing about global simulations. Therefore capturing spot formation in global simulations is perhaps as challenging, or even more challenging, than cracking the convective conundrum. Nevertheless, recent spot formation studies in more idealised simulation setups serve as a guide for the design of future global simulations that aim at achieving this.

All of these developments happen on a background where modelers have started to realize that the holy grail of stellar dynamo simulations – an asymptotic regime where results are independent of resolution or diffusivity – remains elusive, and that the computational cost of adding another data point at a higher resolution is already prohibitive. This begs the question whether it is feasible for everyone to try to beat everyone else in this very difficult task or whether it is better to combine resources for a collaborative effort where the resources of at least a large part of the field are directed in producing the “next generation” transformative simulations.

## Footnotes

1 https://www.amd.com/en/products/cpu/amd-epyc-9654.
